# Capillary pruning couples tissue perfusion and oxygenation with cardiomyocyte maturation in the postnatal mouse heart

**DOI:** 10.3389/fcell.2023.1256127

**Published:** 2023-11-07

**Authors:** Ricardo Santamaría, Javier Cruz-Caballero, Polyxeni Gkontra, Alberto Jiménez-Montiel, Cristina Clemente, Juan A. López, María Villalba-Orero, Jesús Vázquez, Andreas Hutloff, Enrique Lara-Pezzi, Alicia G. Arroyo

**Affiliations:** ^1^ Vascular Pathophysiology Area, Centro Nacional de Investigaciones Cardiovasculares (CNIC), Madrid, Spain; ^2^ Centro de Investigaciones Biológicas Margarita Salas (CIB-CSIC), Madrid, Spain; ^3^ Artificial Intelligence in Medicine Lab (BCN-AIM), Departament de Matemàtiques i Informàtica, Universitat de Barcelona, Barcelona, Spain; ^4^ Cardiovascular Proteomics Lab, Centro Nacional de Investigaciones Cardiovasculares (CNIC), Madrid, Spain; ^5^ CIBER de Enfermedades Cardiovasculares (CIBERCV), Madrid, Spain; ^6^ Myocardial Pathology Area, Centro Nacional de Investigaciones Cardiovasculares (CNIC), Madrid, Spain; ^7^ Institute of Immunology, University Hospital Schleswig-Holstein, Kiel, Germany; ^8^ German Rheumatism Research Centre, A Leibniz Institute, Berlin, Germany

**Keywords:** capillary pruning, postnatal heart, vasodilators, blood flow, oxygenation, metabolism, cardiomyocyte maturation

## Abstract

**Introduction:** Removal of poorly perfused capillaries by pruning contributes to remodeling the microvasculature to optimize oxygen and nutrient delivery. Blood flow drives this process by promoting the intravascular migration of endothelial cells in developing networks, such as in the yolk sac, zebrafish brain or postnatal mouse retina.

**Methods:** In this study, we have implemented innovative tools to recognize capillary pruning in the complex 3D coronary microvasculature of the postnatal mouse heart. We have also experimentally tested the impact of decreasing pruning on the structure and function of this network by altering blood flow with two different vasodilators: losartan and prazosin.

**Results:** Although both drugs reduced capillary pruning, a combination of experiments based on *ex vivo* imaging, proteomics, electron microscopy and *in vivo* functional approaches showed that losartan treatment resulted in an inefficient coronary network, reduced myocardial oxygenation and metabolic changes that delayed the arrest of cardiomyocyte proliferation, in contrast to the effects of prazosin, probably due to its concomitant promotion of capillary expansion.

**Discussion:** Our work demonstrates that capillary pruning contributes to proper maturation and function of the heart and that manipulation of blood flow may be a novel strategy to refine the microvasculature and improve tissue perfusion after damage.

## 1 Introduction

During organism development, primitive vascular networks are immature and need to be refined for proper function (Abe et al., 2019). One of the most relevant processes to this remodeling is capillary pruning, the selective elimination of poorly functional vascular segments to favor the formation of hierarchical networks (Santamaria et al., 2020).

Recent developments in confocal microscopy and image analysis led to the current model for capillary pruning that establishes blood flow as the major determinant of the directed migration against flow of endothelial cells in the poorly perfused vessel towards the adjacent highly perfused vessel as shown in the yolk sac, the 2D stereotyped vasculature of the mouse postnatal retina and in zebrafish brain vessels where the small GTPase Rac1 seems to be relevant (Lucitti et al., 2007; Chen et al., 2012; Udan et al., 2013; Franco et al., 2015). Of note, gradients of blood flow rather than its absolute magnitude appear to be the trigger of pruning events (Santamaria et al., 2020) as supported by computational simulations (Bernabeu et al., 2014), and by the reported effects of vasoconstriction in vessel pruning in pathophysiological angiogenesis in the retina (Lobov et al., 2011; Franco et al., 2016).

Open questions remain about the presence and relevance of pruning in complex vascular networks particularly in physiological conditions. Capillary pruning is proposed to serve for optimizing the distribution of blood flow, and thus oxygen and nutrients, from large vessels to all distal territories, and computational simulations in the postnatal retina vasculature support this idea (Watson et al., 2012), but testing it experimentally remained challenging. Moreover, although excessive capillary pruning seems to underlie reduced vascular density after myocardial infarction (Gkontra et al., 2018) or Alzheimer’s disease (Religa et al., 2013) and capillary pruning has been associated to aging and loss of neuronal activity in the brain (Gao et al., 2020), little is known about the impact of pruning in organ development.

Given the complex 3D nature of the coronary microvasculature, the constant metabolic demand of the heart and the relevance of oxygen in cardiomyocyte maturation (Guo and Pu, 2020), we chose the postnatal mouse cardiac microvasculature as the model to investigate these open questions in the capillary pruning field. In this work we recognize for the first time the occurrence of capillary pruning in the developing coronary microvasculature of the neonatal heart, and we show that this pruning is essential for optimal perfusion and oxygenation of the tissue and, consequently, for adequate maturation and function of cardiomyocytes.

## 2 Materials and methods

### 2.1 Mouse strains

Neonates from wild-type C57BL/6 mice (Jackson Laboratories) were used at postnatal days 1, 7, and 14 (P1, P7, P14). Mice were housed in the animal facility of the National Center for Cardiovascular Research (CNIC) or the Centro de Investigaciones Biológicas Margarita Salas (CIB-Margarita Salas-CSIC) under pathogen-free conditions and in accordance with institutional guidelines. Animal studies were approved by the local ethics committees in accordance with the 2010/63EU directive and recommendation 2007/526/EC on the protection of animals used for experimental or scientific purposes, imposed by Spanish law RD1201/2005 (permit numbers: PROEX 34/13 and PROEX 094.5/22). Neonates from heterozygous Klf2^eGfp/+^ mice (Weinreich et al., 2009) were bred by Dr. Hutloff’s laboratory (Berlin) in the pathogen-free facility of the Deutsches Rheuma-Forschungszentrum (DRFZ), following the protocols of animal welfare and care and with the relevant permits from the German local authorities (Landesamt für Gesundheit und Soziales, permit number: G 0209/19). Tissues (fixed and frozen) were provided for further processing and analysis in our laboratory.

### 2.2 Animal procedures

The vasodilator drugs Losartan and Prazosin (Sigma) were injected daily subcutaneously under the nape of the neck of neonates from P2 to P6 diluted in 75 µL of saline; control mice were injected with 75 µL of saline without drugs. Previous reports had used losartan at 40 mg/kg/day for 6 days (Chauhan et al., 2013; Martin et al., 2022) or prazosin at 6–10 mg/kg/day for 1–2 weeks (Zhou et al., 1998; Williams et al., 2006a; Mandel et al., 2016) to analyze their impact on vascular remodeling. And standard oral therapeutic doses in pediatric patients are slightly higher for losartan (0.7 mg/kg/day up to 50 mg/day) compared to prazosin (0.1–0.5 mg/kg/day up to 20 mg/day). We therefore chose to inject the same two doses of each vasodilator, a low dose of 10 mg/kg/day and a higher dose of 25 mg/kg/day, close to the range used experimentally and far from rodent toxicity doses (https://go.drugbank.com/drugs/DB00678 and https://go.drugbank.com/drugs/DB00457). The pups were sacrificed at 7 days of age. For perfusion and vascular integrity experiments, prior to sacrifice, pups were anaesthetized and 10 µL fluorescent dextran and 10 µL isolectin B4 (5 min apart) were injected retro-orbitally into the left and right eye, respectively. After a further 5 min, the mice were euthanized. The Hypoxyprobe Omni-Kit was used to detect hypoxia. Pimonidazole was injected intraperitoneally at a concentration of 60 mg/kg diluted in 100 µL saline into P7 neonates, previously treated with saline or vasodilator drugs, and the mice were sacrificed 30 min later.

### 2.3 Tissue processing and immunofluorescence staining

The pups were sacrificed by decapitation and the hearts perfused with cold PBS before removal. Hearts were immediately fixed in 4% paraformaldehyde (PFA) overnight at 4°C. Subsequently, hearts were immersed until they stopped floating sequentially in 15% and 30% sucrose before freezing at −80°C embedded in OCT. OCT-embedded hearts were sectioned on the Leica CM1850 cryostat at 35 µm. Hearts from pimonidazole-injected mice were embedded in paraffin, after fixation with 4% PFA. Thin 5 µm tissue sections were cut on a Leica RM2245 semi-automated microtome, dewaxed, rehydrated and antigen retrieved using citrate buffer (10 mM sodium citrate, 0.05% Tween 20, pH6) before staining for hypoxia or for proliferation. To obtain retinas, eyes were removed from mouse pups and gently fixed with 4% PFA on ice for 1 hour and subsequently, retinas dissected as described (Pitulescu et al., 2010). After dissection, retinas were re-fixed for 1 h with 4% PFA at room temperature and stored at 4°C in PBS until staining. Immunofluorescence staining of thick heart sections was performed in flotation and of whole-mount retinas following previous protocols (Pitulescu et al., 2010). Thick tissue sections were blocked and permeabilized in a solution of 5% BSA with 2.5% goat serum in PBS-0.2% Triton X100 (PBS-T) for 1 hour at room temperature and then incubated with the corresponding primary antibodies diluted in a solution containing 2.5% BSA and 1.25% goat serum in 0.2% PBS-T overnight at 4°C. After three washes of 30 min with 0.3% PBS-T, followed by another wash in PBS, thick sections were incubated with the corresponding secondary antibodies coupled to fluorochromes and diluted in the same solution as the primary antibodies overnight at 4°C. After incubation, the sections were washed again 3 times with 0.3% PBS-T, followed by a final wash with PBS for 30 min and the samples were mounted with Fluoromount. Whole retinas were blocked and permeabilized in a solution of 5% BSA with 1.25% goat serum in PBS-0.2% Triton X100 (PBS-T) for 1 hour at room temperature and then incubated with the corresponding primary antibodies diluted in the same solution overnight at 4°C. In cases where isolectin B4 (IB4) was used as the primary reagent, both blocking and incubation with the primary antibody were performed in Pblec (PBS containing 1mM MgCl_2_, 1mM CaCl_2_ and 0.1mM MnCl_2_). After four washes of 20 min with 0.3% PBS-T and 2.5% BSA, followed by another wash in PBS, retinas were incubated with the corresponding secondary antibodies coupled to fluorochromes and diluted in the same solution as the primary antibodies for 1 h at room temperature. After incubation, the retinas were washed again 3 times with 0.3% PBS-T, followed by a final wash with PBS for 30 min and the samples were mounted with Fluoromount. Immunofluorescence on thin tissue sections was performed in a wet chamber using the Dako Pap pen with a protocol similar to that for thick sections, but with 10-min washes and incubation of secondary antibodies for 1 h at room temperature. Different combinations of primary antibodies or reagents were used for visualizing the different processes and events, in particular for: pruning events, anti-ICAM2 or IB4 (vascular lumen), anti-Collagen IV (basement membrane), and anti-ERG (endothelial nuclei); blood flow sensing, anti-GFP (for KLF2), anti-ERG (endothelial nuclei) and anti-SMA (for smooth muscle cells); vascular permeability and perfusion, dextran and biotinylated-IB4 with streptatividin; tissue hypoxia, anti-pimonidazole adduct (after antigen retrieval with citrate buffer pH6) and biotin-IB4; and cardiomyocyte proliferation, anti-Ki-67, anti-cTnT (cardiomyocytes) and DAPI or Hoechst 33342 (nuclei).

### 2.4 Confocal microscopy and image acquisition

For pruning events images were obtained by mosaic scanning of the entire heart, with sections acquired every 2 µm using the Nikon A1R confocal microscope with ×20 air objective. For hypoxia detection, images were obtained by mosaic scanning of the entire heart with sections acquired every 2 µm using the Leica SP8 (for P1, P7, and P14 hearts) or the Nikon A1R (for P7 hearts treated with saline, losartan or prazosin) confocal microscope with ×20 air objective. Retina imaging was performed by mosaic scanning of the entire retina using the Nikon A1R confocal microscope with the ×20 air objective. For laminar flow sensing and permeability/perfusion assessment, images from thick heart sections were acquired by mosaic scanning every 2 µm on the Zeiss LSM700 confocal microscope using the ×20 oil objective. For cardiomyocyte proliferation, images were acquired by mosaic scanning of the heart on a SP8 Leica confocal microscope with the ×20 air objective.

### 2.5 Image analysis

For *3D capillary pruning analysis in the mouse postnatal hearts*, images of confocal microscopy sections were processed and reconstructed in 3D in Imaris v9.5 software (Bitplane Corp) with maximum intensity projection mode at rendering quality. Capillary pruning events were manually quantified as empty sleeves in the 3D tissue reconstruction that could be moved freely to encompass the entire volume. Capillary pruning events were quantified either in the entire ventricular wall or septum of P1, P7, and P14 hearts or in 5 volumes of interest (350 × 350 × 35 µm) selected in the myocardium of the left ventricular free wall in hearts from P7 treated-mice. For *endothelial density maps*, a macro was implemented in ImageJ software (Schindelin et al., 2012), modifying the previous BioVoxxel plugin (Brocher, 2014), based on ‘neighbor analysis’. A mean intensity filter and watershed function were applied to maximum intensity projection (MIP) images obtained from three Erg-stained sections spanning a total of 6 µm to segment individual endothelial cell nuclei and the number of neighbors of each endothelial nucleus within 32 µm radius was quantified. The histogram with the distribution of the number of neighbors was obtained and a color code was assigned to each endothelial nucleus according to its number of neighbors. *In the mouse postnatal retina,* capillary pruning events were quantified manually as empty sleeves and the number of endothelial cells as Erg-positive particles in ImageJ, and vascular density calculated with the open software Angiotool (Zudaire et al., 2011).

The *cardiac 3D vasculature* of P1, P7 and P14 hearts was analyzed in Imaris v9.5 (Bitplane Corp.) to quantitate the vascular volume density (by segmentation and surface rendering) and endothelial cell number (detection tool of rounded volumes). The *cardiac 3D vasculature* of P7 hearts treated with saline or vasodilators was analyzed by the algorithm previously implemented by our laboratory (Gkontra et al., 2018) adapted to ICAM2-stained vasculature. This algorithm performs the automatic segmentation of the microvasculature by the ‘multi-scale multilevel thresholding’ approach and reconstructs and skeletonized the vascular network. The software quantifies parameters related to fractal analysis, vascular segment angio-architecture, and tissue oxygenation (Gkontra et al., 2015; Gkontra et al., 2018) in 3D. Similar to capillary pruning events, these parameters were quantified in the same 5 volumes of interest (350 × 350 × 35 µm) selected in the myocardium of the left ventricular wall for pruning events quantification.

*Vascular perfusion* in the entire left ventricle wall was quantified in ImageJ as the percentage of ICAM2-positive vasculature occupied by i. v. injected IB4:

$$Percentage\;of\;vasculature\;perfused=\frac{Isolectina\;B4\;positive\;area}{ICAM2\;positive\;area}\cdot 100$$

*Vascular integrity* was calculated by subtracting ICAM2-positive area from the image and calculating the percentage of extravascular dextran in the corresponding tissue:

$$Percentage\;of\;extravascular\;dextran=\frac{Positive\;dextran\;area}{Total\;tissue\;area}\cdot 100$$

For quantification of *tissue hypoxia*, pimonidazole-stained area was converted into 8-bitmap (P1, P7 and P14 hearts) or 16-bitmap (P7 treated hearts) images in ImageJ displaying the signal intensity from 0 to 4095 for each pixel of the image. The mean intensity of all tissue-contained pixels was quantified, and the frequency distribution histogram was obtained. In addition, a pseudocolor palette was applied to the images for better visualization of the global and regional hypoxic areas.

*Cardiomyocyte proliferation* was quantified in 5 fields of 350 × 350 μm^2^ selected in the myocardium of the left ventricular wall for each mouse in paraffin-embedded stained thin sections by manual identification of Ki-67-positive nuclei (Hoechst positive), manual assignment of cardiac troponin (cTnn) positivity and normalization to the area occupied by cardiac tissue. Quantification was performed by two independent observers, one of whom was blinded to sample treatment.

### 2.6 PAS histochemistry

Paraffin-embedded thin sections from neonatal hearts were processed, stained for Periodic acid-Schiff (PAS) and images acquired at the Histology Unit from National Center for Cardiovascular Research (CNIC, Madrid). These images were converted to binary mode, a mask of the myocardial tissue was manually generated and quantification of the percentage of PAS-positive stained area was performed with a plug-in in ImageJ (Santamans et al., 2021), adjusting the RGB value threshold to the PAS positive signal in the control tissues.

### 2.7 Transmission electron microscopy and mitochondria analysis

Hearts were extracted from mouse neonates after cold PBS perfusion and fixed in a solution of 50 mL PBS containing 4% PFA and 2% glutaraldehyde for 2 h at room temperature. Fixed hearts were then embedded in resin (Durcupan ACM Fluka, Sigma-Aldrich) and ultrathin sections were cut and counterstained at the Electron Microscopy Unit at the CIB. Sections were imaged using a transmission electron microscope (JEOL JEM-1230, 120 Kv, Jeol Ltd. Tokyo, Japan) and recorded with a digital camera CMOS TVIPS TemCam-F416, 16 mega pixels (16 × 16 µm of pixel size) at the indicated magnifications. Hearts from neonatal mice treated with saline, losartan or prazosin were visualized and selected images used to quantitate morphological parameters of at least 300 mitochondria per condition by manually drawing in ImageJ software.

### 2.8 Proteomic analysis based on isobaric labelling with TMT (tandem-mass-tag) reagents

Fresh hearts were dissected from mouse P7 neonates after perfusion with PBS, the atria were removed and then immediately frozen in liquid nitrogen and stored at −80°C. A total of 11 samples were analyzed with 3 mice treated with saline, 4 treated with Losartan and 4 treated with Prazosin from 3 independent litters. Protein extracts were obtained by mechanical disintegration of frozen hearts in FastPrep tubes with 50mM Tris-HCl pH7.5% and 2% SDS, with three rupture cycles (6,000 rpm/s for 1 min each), and after removal of debris by centrifugation at 12,000 g/10 min, protein extracts were quantified using the DirectDetect equipment (Millipore). Proteins (120 μg) were then digested by the FASP method (Wisniewski et al., 2009), and peptide TMT labeling (TMT11plex; Thermo Scientific) was performed according to the manufacturer’s instructions. After labelling and mixing of samples, removal of excess labelling reagents and desalting was accomplished in HLB cartridges (Oasis, Waters Corporation, Milford, MA, United States). For increasing the proteome coverage, TMT-labelled peptides were fractionated on C18 reversed phase columns (High pH Fractionation Kit; Thermo Scientific) into 6 fractions following the supplier’s protocol. High-resolution analysis of TMT-labelled peptides was carried out on an Easy nLC 1000 nano-HPLC system (Thermo Scientific, San Jose, CA, United States) coupled to a Tribrid Orbitrap mass spectrometer (Orbitrap Fusion, Thermo Scientific). Peptides were suspended in Buffer A (0.1% formic acid) and then loaded onto a pre-column (PepMap100 C18 LC 75 µm ID, 2 cm, Thermo Scientific) and separated on-line on a NanoViper PepMap100 C18 LC analytical column (75 µm ID, 50 cm, Thermo Scientific) in a continuous gradient consisting of 5%–32% B for 240 min and 32%–90% B for 5 min (B = 100% acetonitrile, 0.1% formic acid) at 200 nL/min. Each MS run consisted of enhanced FT-resolution spectra (60,000 resolution) in the 400–1,500 m/z range followed by data-dependent MS/MS spectra of the most intense parent ions acquired during the chromatographic run. The AGC target value in the Orbitrap was set to 200,000. Fragmentation was performed at 36% normalized collision energy with a target value of 50,000 ions, 30,000 resolution, 120 m injection time, and 40 s dynamic exclusion. For peptide identification the MS/MS spectra were searched with the Sequest HT algorithm (Tabb, 2015) implemented in Proteome Discoverer 2.5 program (Thermo Fisher Scientific). Against an Uniprot database containing all sequences from mouse (UniProt_May 2021; 25332 entries). Searching parameters were selected as follows: trypsin digestion with 2 maximum missed cleavage sites, precursor and fragment mass tolerances of 2 Da and 0.03 Da, respectively, carbamidomethyl cysteine, and TMT modifications at N-terminal and Lys residues as fixed modifications, and methionine oxidation was selected as dynamic modification. Peptide identification was performed using the probability ratio method (Martinez-Bartolome et al., 2008). False discovery rate (FDR) was calculated using an inverted database and the refined method (Navarro and Vazquez, 2009), using a cut-off value of 1% FDR with an additional filtering for precursor mass tolerance of 15 ppm (Bonzon-Kulichenko et al., 2015). The data were analyzed using the weighted spectrum, peptide and protein statistical model WSPP (Navarro et al., 2014) with the SanXoT package (Trevisan-Herraz et al., 2019). This model expresses quantitative protein values as standardized log2ratios (Zq). Differences in protein abundance between groups were analyzed by Student’s t-test. Analysis of functional category alterations produced by coordinated protein behavior was performed based on the Systems Biology Triangle model (Garcia-Marques et al., 2016), using a FDR threshold of 0.05. Functional categories were obtained from Gene Ontology, Ingenuity Pathway Analysis, Kegg and David Pathways databases.

### 2.9 Echocardiography and electrocardiogram analysis

Transthoracic echocardiography was performed under slight anesthesia (0.5%–2.0% isoflurane in 100% O_2_) using a high-frequency ultrasound equipment (Vevo 2100, Visualsonics Inc., Canada) with the support of the CNIC Advanced Imaging Unit, which quantified several structural and functional parameters (Supplementary Table S3). Coronary artery diameter and blood flow velocity were measured in systole and diastole. The differential (Δ = P7-P1) was plotted for the magnitude parameters and the percentage differential (%Δ) was plotted as the % variation between P1 and P7 for the functional parameters. The deformation of the left ventricular posterior wall in relation to contractility was calculated with the following formula (Heimdal et al., 1998; Voigt and Flachskampf, 2004):

$$Strain=\frac{\left(LVW\;thickness\;in\;systole\right)-\left(LVW\;thickness\;in\;diastole\right)}{LVW\;thickness\;in\;systole}\cdot 100$$

To assess cardiac electrophysiological activity, electrocardiogram was performed in anesthetized mice using standard bipolar and unipolar limb leads (MP36 system, BIOPAC Systems, Inc., United States) at P7 in basal conditions for 3 min (Supplementary Table S3). Electrocardiograms were analyzed using Acqknowledge 4.1.1. Correction of the duration of QRS complex and QT interval by the heart rate was performed according to the following equations (Mason et al., 2019): *QRSc* = *QRS* + 0.0125 ∙ (1 − *RR*) and *QTc = QT + 0.0154 ∙ (1 − RR)* where RR is the interval between R waves in ms.

### 2.10 Cadiomyocyte isolation and analysis

Cardiomyocytes were isolated from P7 neonatal hearts as described previously (Mollova et al., 2013). Fresh hearts were cut in small pieces, fixed in 4% PFA for 2 h, and digested with collagenase B (1.8 mg/mL) and D (2.4 mg/mL) at 37°C overnight in agitation. For nucleation counts, the cardiomyocytes were incubated with blocking solution containing PBS, 0.3% Tx100, 5% BSA and 5% NGS for 1 h at room temperature, mouse anti-cTnn CT3 antibody (1:10; DSHB), and goat anti–mouse-488 (1:400; Invitrogen) and DAPI (1:1.000; EMD Millipore) for 1 h at room temperature. Stained cardiomyocytes were dropped on a slide and mount with Fluoromount. Images were acquired with a Leica SP8 or SP5 confocal microscope with a ×40 oil objective. At least 1000 cardiomyocytes per condition were analyzed for percentage of binucleation/mononucleation. Individual cardiomyocytes (between 100-250 per condition) were also segmented in ImageJ for quantification of morphological features (area and elliptical factor).

### 2.11 Statistical analysis

All analyses and graphs shown were performed with GraphPad Prism 8 software (GraphPad Software, La Jolla, United States). In cases where two groups with a normal distribution of data according to the Shapiro-Wilk test were compared, the two-tailed Student’s *t*-test was used. For more than two groups, if at least one of the data distributions was normal according to the Shapiro-Wilk test, the one-way ANOVA test with Tukey’s *post hoc* corrections for multiple testing was performed, unless otherwise indicated. If no distribution was normal, the Kruskal-Wallis test was performed and multiple comparisons were corrected with Dunn’s method. To compare proportions, Fisher’s exact test was used. The interrelation of parameters was analyzed by computing Pearson’s correlation coefficients. In all cases, a significant difference was considered to exist when *p* ≤ 0.05, and significance was represented as follows: *p ≤ 0.05, **p ≤ 0.01, ***p ≤ 0.001 and ****p ≤ 0.0001.

List of antibodies and other reagents for tissue staining.

**Table:** 

Antibody/reagent	Company	Reference	Dilution
Collagen IV	Serotec	2150–1470	1:150
DAPI	Thermo Scientific	10116287	1:5000
ERG-647	Abcam	Ab196149	1:150
GFP	Abcam	Ab13970	1:150
ICAM2	Pharmingen	553325	1:150
IsolectinB4	Vector	B-1205	1:50
Ki67	Abcam	Ab16667	1:100
Pimonidazole	Hypoxyprobe	Pab2627AP	1:100
SMA	Sigma	A5228	1:200
Streptavidin-647	Fisher	10308062	1:500
TNNT2	DSHB	CT3	1:10

List of other reagents.

**Table:** 

Reagent	Company	Reference
Losartan	Sigma	61188
Prazosin	Sigma	P7791
Pimonidazole	Hypoxyprobe	Omni Kit HP3-100Kit
Dextran	MPBiomedicals	0216011010
Fluoromount-G	SouthernBiotech	0100 01

## 3 Results

### 3.1 Capillary pruning occurs during remodeling of the coronary microvasculature in the postnatal mouse heart

**FIGURE 1:**
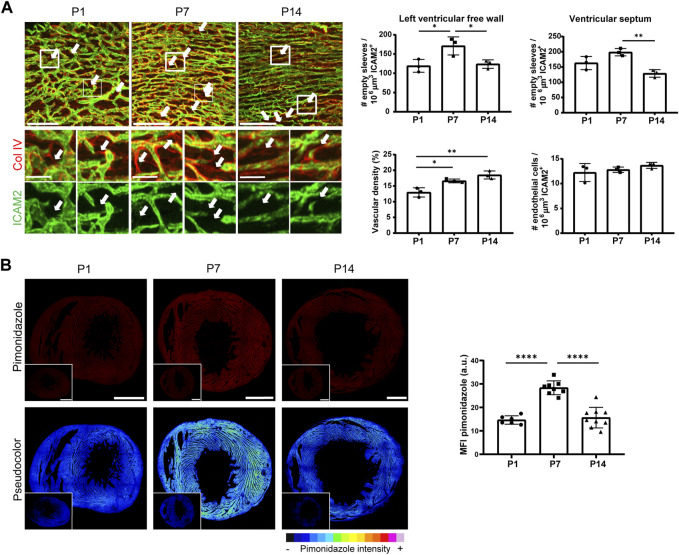
Vascular remodeling occurs by capillary pruning in the mouse postnatal cardiac microvasculature and correlates with changes in tissue oxygenation. **(A)**, Confocal microscopy maximum intensity projections (MIP) of ICAM2 (green) and collagen IV (red) staining (left) and quantification of empty sleeves, vascular density and endothelial cells in the LVFW myocardium and of empty sleeves in the septum (right) in P1, P7 and P14 mouse hearts. Arrows indicate ‘empty sleeves’. Scale bar, 100 μm and 25 µm in the magnifications. **(B)**, Confocal microscopy MIP of hypoxyprobe signal (red) and pseudocolor visualization (left) in the LVFW after conversion to 8-bitmap images in ImageJ and quantification of the mean intensity fluorescence (MFI) of the hypoxyprobe (right) in P1, P7 and P14 mouse hearts. Insets show negative controls from mice not injected with pimonidazole. Scale bar, 500 µm. See also Supplementary Figure S1. Bar graphs in A and B show individual values and means ± S.D. and the data were compared by one ANOVA test with Tukey’s multiple comparisons, *p ≤ 0.05, **p ≤ 0.01, ****p ≤ 0.0001.

We first observed that the coronary microvasculature changes during the first weeks after birth from a dis-organized network observed at postnatal stage 1 (P1) to a better-patterned and hierarchized network by P14 (Figures 1A; Supplementary Figure S1A). This observation together with expected changes in cardiac blood flow after birth (Rudolph, 1970), prompted us to hypothesize that capillary pruning could be a contributor to remodeling of the postnatal coronary microvasculature. Given the intricate 3D nature of this network, we first implemented a pipeline for image acquisition and analysis that could allow the identification of capillary pruning events if present. We stained 35 µm-thick sections from postnatal hearts at different stages, 3D rendered about 18 planes and quantitated empty sleeves (basement membrane COL IV+/endothelial cells ICAM2-), the hallmark of pruning events (Franco et al., 2015), by means of Imaris^®^ software (Supplementary Figure S1B; see M&M section for details).

We focused on the microvasculature of the left ventricular free wall (LVFW) myocardium for the majority of this study by quantifying five representative 3D volumes of 350 μm × 350 μm x 35 µm. We found pruning events in the order of hundreds per mm^3^ of coronary microvasculature already in P1, with a maximum in P7 and a subsequent decrease at P14 (Figure 1A). Pruning of capillaries was also present in the microvasculature of the septum at similar kinetics and higher abundance (Figure 1A), which may suggest subtle differences depending on the cardiac territory. This dynamic remodeling occurred in parallel with a progressive increase in vascular density from P1 to P14 but without changes in relative endothelial cell abundance within the vasculature of the LVFW myocardium (Figure 1A). Therefore, we implemented a custom-made macro in ImageJ (https://imagej.nih.gov/ij/) (Schindelin et al., 2012) based on the ‘number of neighbors’ plugin (Brocher, 2014) (Supplementary Figure S1C, see M&M for more details), and observed that for a given number of endothelial nuclei, the myocardium at P7 showed a more heterogeneous distribution of endothelial cells, with areas of higher density than at P1 and P14 (Supplementary Figure S1D). These findings support that pruning events occurred in parallel with endothelial cell rearrangements in the postnatal heart microvasculature.

We then assessed whether the better organized pattern of the coronary microvasculature observed at P14 (Figure 1A; Supplementary Figure S1A) correlated with a better capacity of the network to distribute blood flow to the distal territories. For this purpose, we analyzed tissue oxygenation by pimonidazole injection and observed that the intensity of this hypoxyprobe was highest at P7, especially in some packed and dense areas of the myocardium, and decreased at P14, suggesting improved tissue oxygenation at this stage (Figure 1B). These data show that maximal remodeling of the cardiac microvasculature by capillary pruning at P7 precedes the achievement of a more efficient coronary network at P14.

### 3.2 The angiotensin receptor 1 (ATR1) inhibitor losartan reduces capillary pruning in the mouse postnatal myocardium

We then set out to reduce capillary pruning events and analyze the impact on the structure and function of the coronary microvascular network. First, we tested the effects of the vasodilator losartan, an angiotensin receptor 1 inhibitor widely used as an antihypertensive drug (Nehme et al., 2019), given previous reports that angiotensin II increased the number of pruned segments in the postnatal mouse retina (Franco et al., 2016) and that the angiotensin converting enzyme inhibitor captopril reduced vascular pruning in a model of retinal pathology (Lobov et al., 2011). We subcutaneously injected two doses of 10 and 25 mg/kg/day of losartan into neonates from P2 to P6 (Supplementary Figure S2A) in the range of those previously used in mice (Lobov et al., 2011). Although there was no effect on body weight, heart weight decreased slightly at both doses and the heart weight/body weight ratio decreased significantly at the low dose of losartan, suggesting an impact on heart growth in this condition (Supplementary Figure S2B).

**FIGURE 2:**
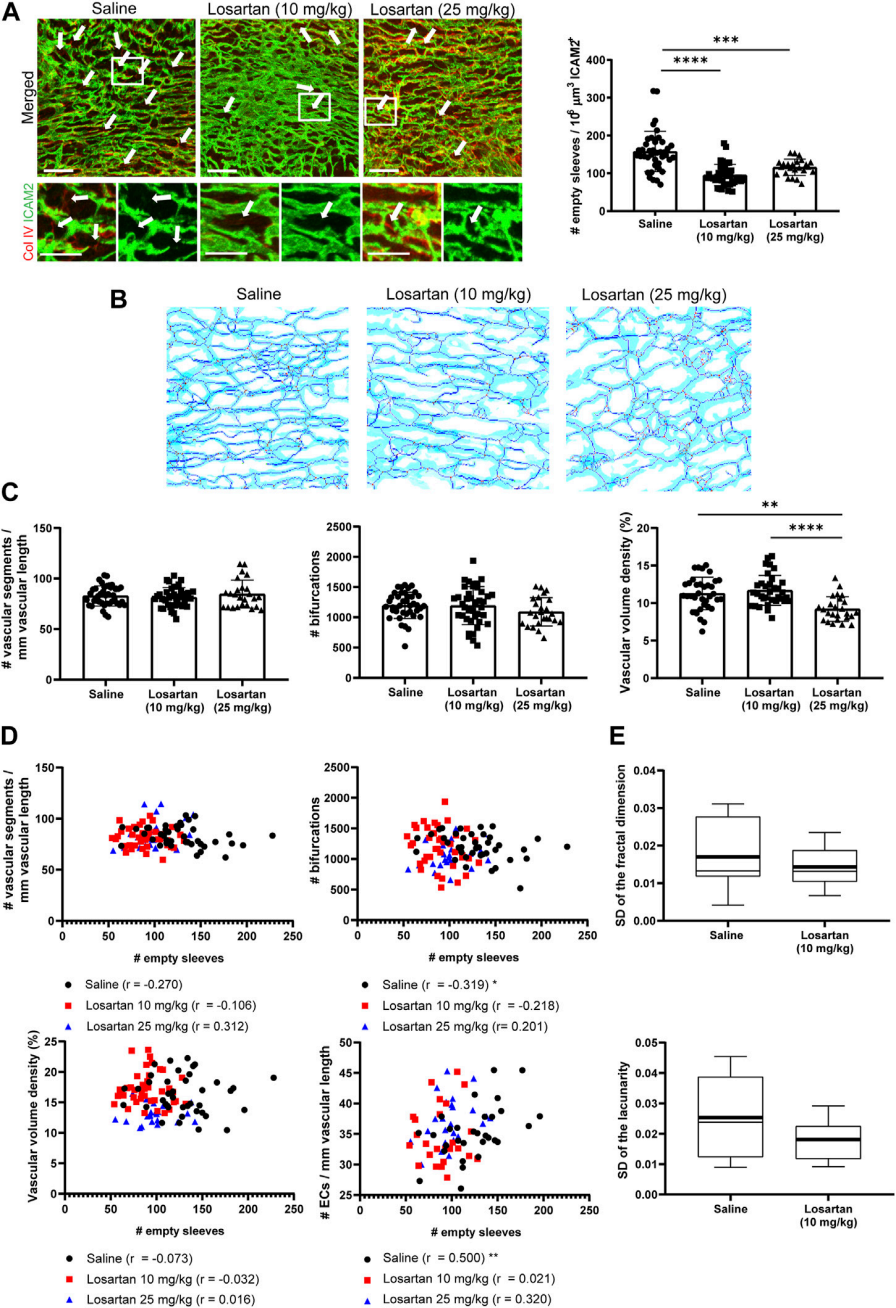
The vasodilator losartan decreases the number of pruning events in P7 mouse hearts with a slight impact on vascular density and network heterogeneity. **(A)**, Confocal microscopy MIP of ICAM2 (green) and Col IV (red) staining (left) in P7 hearts from mice treated with saline or two different doses of losartan from P2 to P6 and quantification of pruning events (right). Arrows indicate ‘empty sleeves’. Scale bar, 50 µm. **(B)**, ICAM2-based skeletons of the heart microvasculature in mice treated as in A using our previously published algorithm (Gkontra et al., 2015; Gkontra et al., 2018). Light blue, dark blue, red and green is used for the microvasculature, skeleton, branching and ending nodes, respectively. **(C)**, Quantification graphs of the number of segments and bifurcations and vascular volume density in losartan-treated mice and controls. **(D)**, Representation of the number of pruning events *versus* the number of segments, bifurcations, vascular volume density and endothelial cells per vascular length in P7 hearts from mice treated as in A. **(E)**, Box plots of the standard deviation of fractal dimension and lacunarity values in the 5 volumes analyzed for each heart from mice treated as in A. Bar graphs in A and C show individual values and means ± S.D. and the data were compared by one ANOVA test with Tukey’s multiple comparisons, *p ≤ 0.05, **p ≤ 0.01, ****p ≤ 0.0001. Graphs in D show Pearson’s correlation coefficients “r” and statistical significance of the correlation, *p ≤ 0.05, **p ≤ 0.01. Box plots in D show the mean (thick line) and median (thin line) of the data values. See also Supplementary Figure S2.

We first confirmed that this ATR1 inhibitor reduced capillary pruning and increased vascular density in the canonical model of the postnatal mouse retina at P7 at the highest dose (Supplementary Figures S2C, S2D) (Lobov et al., 2011). Of interest, both doses of losartan decreased the number of capillary pruning events in the coronary microvasculature of the LVFW from P7 mice (Figure 2A). We then analyzed graph-based parameters of the 3D network with the MatLab algorithm previously implemented by our laboratory (Gkontra et al., 2018) (Supplementary Table S1, Figure 2B, and Supplementary Movies S1–S3). Although reduced pruning would primarily affect the abundance of individual segments, no significant differences in the number of segments or bifurcations were detected in the cardiac microvasculature of losartan-treated mice compared with saline-treated controls (Figure 2C). The main impact observed with low doses of losartan was an increase on segment diameter and also a decreased tortuosity, probably related to its vasodilation effects on small size vessels, and thus a slight but not significant increase in vascular volume density (Supplementary Table S1). Given the low number of pruning events (100–150) relative to the total number of segments (around 2,000-3,000) in each tissue volume (approximately 4%) and taking into account the short-lived nature and dynamics of these events in likely conjunction with the parallel formation of new sprouts, as recently demonstrated during postnatal vascular remodeling of the skin (Kam et al., 2023) a reduction in pruning events at a defined time-point may not affect the averaged segment-related parameters, but still contribute to network reshape. Accordingly, we observed in each analyzed tissue volume of the control hearts that there was no relationship of the abundance of capillary pruning events with vascular volume density but that it was inversely related to the number of vascular segments and the number of bifurcations (in this case significantly) and also positively and significantly related to the number of endothelial cells per vascular length, supporting that pruning contributes to the number of segments and bifurcations and to endothelial cell rearrangements in the network but that other factors have more weight in determining the vascular volume density (Figure 2D). Interestingly, similar correlations between pruning events and the number of segments and bifurcations were observed in hearts treated with low doses of losartan, suggesting that the influence of this vasodilator on the network is mainly due to reduced capillary pruning (Figure 2D). In contrast, Pearson’s correlation coefficients in hearts treated with high-dose losartan diverged from those in mice treated with saline or low-dose losartan, suggesting that the vascular changes in this case are related to effects other than decreased segment pruning (Figure 2D). We therefore proceeded with the low dose of losartan for further functional analysis, given that the two doses induced a similar reduction in the number of pruning events, that the low dose impacted the entire microvasculature (not just vascular volume density which does not appear to be as sensitive to pruning changes) in a manner consistent with a pruning contribution and also to minimize possible side effects that the higher dose might have on other cell types or processes.

We then examined the impact of low-dose losartan-mediated pruning reduction on network heterogeneity. For this purpose, we quantified the fractal dimension (morphological complexity) and lacunarity (heterogeneity of gap distribution) in each of the five volumes analyzed for pruning with an *ad hoc* algorithm previously implemented in our laboratory (Gkontra et al., 2015; Gkontra et al., 2018) and calculated the coefficient of variation (standard deviation) of these parameters between these different volumes of the same heart (Figure 2E; Supplementary Table S1). The coefficient of variation of fractal dimension and lacunarity reached the highest percentile values in saline-treated control hearts and, moreover, the mean values of the coefficients in these hearts tended to be higher than those in hearts treated with low doses of losartan, suggesting greater heterogeneity of the vascular network in control mice (Figure 2E).

### 3.3 The α1-adrenergic receptor inhibitor prazosin also reduces capillary pruning but changes the structure of the postnatal coronary microvasculature differently

We sought to validate the effects of losartan with another vasodilator, prazosin, an α1 adrenergic receptor antagonist. We injected prazosin subcutaneously from P2 to P6 at 10 or 25 mg/kg/day (Supplementary Figure S3A), doses close to those previously used to induce capillary splitting in mouse or rat skeletal muscle (Zhou et al., 1998; Williams et al., 2006b), Prazosin treatment slightly reduced body and heart weight, with no significant difference in heart weight/body weight ratio at any of the prazosin doses (Supplementary Figure S3B).

**FIGURE 3:**
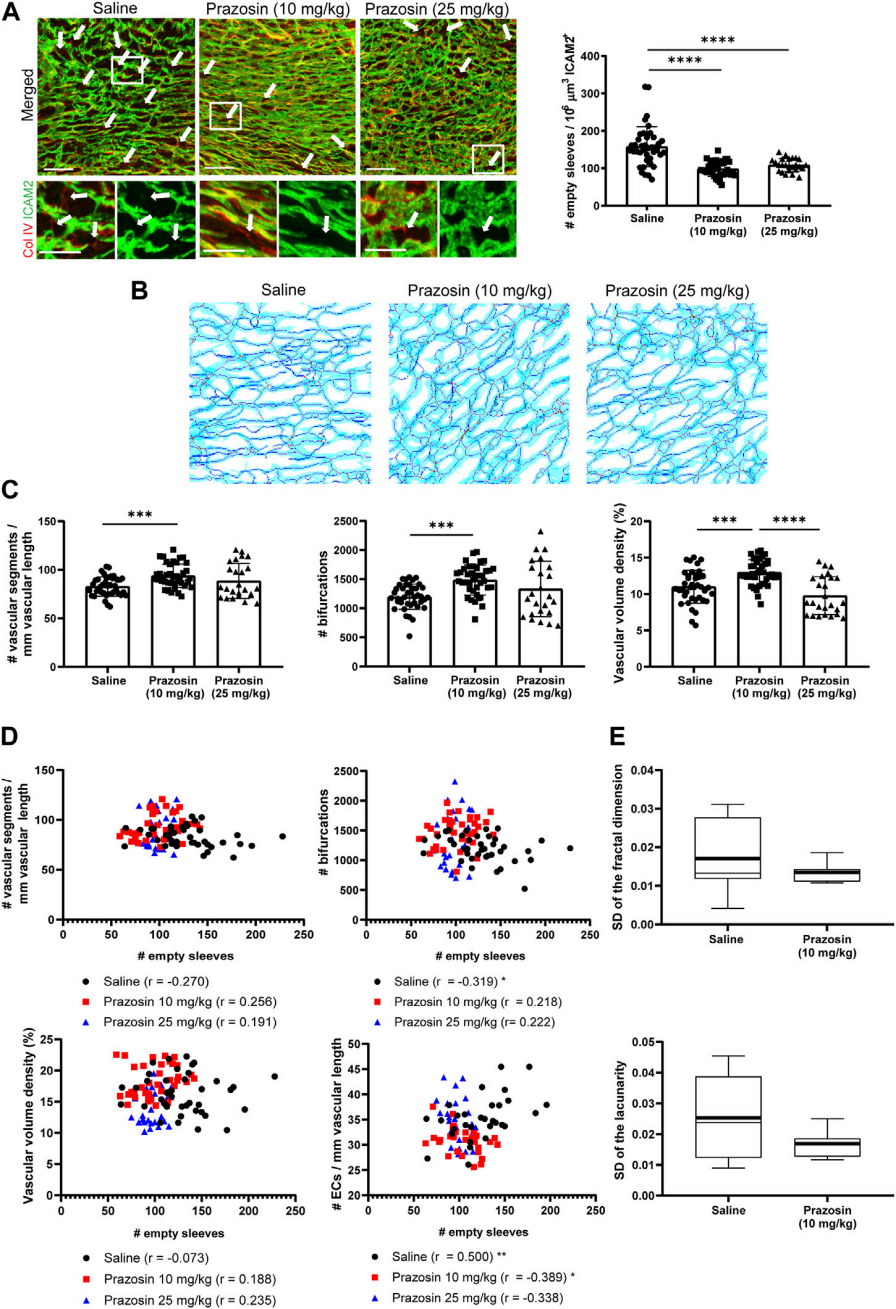
The vasodilator prazosin reduces the number of pruning events in P7 mouse hearts with higher vascular density and lower network heterogeneity. **(A)**, Confocal microscopy MIP of ICAM2 (green) and Col IV (red) staining (left) in P7 hearts from mice treated with saline or two different doses of prazosin from P2 to P6 and quantification of pruning events (right). Arrows indicate “empty sleeves”. Scale bar, 50 µm. **(B)**, ICAM2-based skeletons of the heart microvasculature in mice treated as in A using our previously published algorithm (Gkontra et al., 2015; Gkontra et al., 2018). Light blue, dark blue, red and green is used for the microvasculature, skeleton, branching and ending nodes, respectively. **(C)**, Quantification graphs of the number of segments and bifurcations and vascular volume density in prazosin-treated mice and controls. **(D)**, Representation of the number of pruning events *versus* the number of segments, bifurcations, vascular volume density and endothelial cells per vascular length in P7 hearts from mice treated as in A; Pearson’s correlation coefficients ‘r’ and statistical significance * are indicated. **(E)**, Box plots of the standard deviation of fractal dimension and lacunarity values in the 5 volumes analyzed for each heart from mice treated as in A. Bar graphs in A and C show individual values and means ± S.D. and the data were compared by one ANOVA test with Tukey’s multiple comparisons, *p ≤ 0.05, **p ≤ 0.01, ****p ≤ 0.0001. Graphs in D show Pearson’s correlation coefficients “r” and statistical significance of the correlation, *p ≤ 0.05, **p ≤ 0.01. Box plots in D show the mean (thick line) and median (thin line) of the data values. Images and values for control mice treated with saline are the same than in Figure 2 since the experiments were run in parallel but data obtained with the two vasodilators are presented separately for clarity. See also Supplementary Figure S3.

Prazosin significantly decreased the number of pruning events in P7 retinas at the high dose (Supplementary Figures S3C, S3D), whereas in the coronary microvasculature it reduced the abundance of pruning events at both doses (Figure 3A), similar to losartan treatment. However, the impact of low-dose prazosin on the structural parameters of the coronary vasculature was different from that of losartan (Supplementary Table S1, Figure 3B, and Supplementary Movies S1, S4, S5), since it did not impact segment diameter but induced a significant increase in the number of vascular segments, branching nodes, bifurcations, trifurcations and also in vascular volume density compared with saline (Figure 3C; Supplementary Table S1). Moreover, in volumes from prazosin-treated hearts, Pearson’s correlation coefficients between the number of pruning events and the number of segments, bifurcations, vascular volume density and endothelial cells per vascular length were opposite to that of the controls (Figure 3D), suggesting that the observed impact on the microvasculature was not solely due to reduced capillary pruning, as in the case of the low dose of losartan (Figure 2). Similar to losartan, we proceeded with the low dose of prazosin for subsequent analysis, given that although the two doses induced a similar reduction in the number of pruning events, the high dose of prazosin significantly reduced vascular density compared to the low dose (Figures 3A, 3C, 3D) and also to minimize the potential side effects of the higher dose. Finally, the coefficients of variation of fractal parameters in the quantified cardiac volumes tended to be larger in control hearts than in hearts treated with low doses of prazosin, suggesting greater heterogeneity of the network (Figure 3E).

We sought to delve deeper into the distinctive effects of the two vasodilators by focusing for the remainder of the analysis on the low doses, as the high doses appeared to affect processes beyond capillary pruning (Figures 2, 3). Although the low doses of both vasodilators reduced capillary pruning, prazosin induced distinct microvascular changes that did not simply correlate with such decrease (Figure 3D). We assessed whether this might be related to different impact of vasodilators on blood flow using a Klf2-GFP transgenic mouse model, since the expression of the transcription factor Klf2 in endothelial cells is sensitive to changes in blood flow (Dekker et al., 2002). Low doses of losartan or prazosin tended to increase the percentage of Klf2-GFP-positive endothelial cells in the overall vascular network, but not in the large SMA + vessels, where they decreased it, significantly in the case of losartan, compared with control mice (Supplementary Figure S4A). These findings suggest that both vasodilators similarly alter blood flow in the coronary vasculature, which may underlie their reduction of capillary pruning. Alternatively, the significant increase in the number of segments (particularly those of small diameter), bifurcations, trifurcations and higher order nodes (Esteban et al., 2020), together with the reduction in the number of endothelial cells per vascular length (Supplementary Table S1), support that low dose prazosin may be enhancing the capillary splitting that normally occurs in the heart at this stage (van Groningen et al., 1991) as it does in mouse and rat skeletal muscle (Zhou et al., 1998; Williams et al., 2006b) and in contrast to losartan, in which none of these vascular parameters related to splitting appeared to be affected (Supplementary Table S1).

### 3.4 Reduction of capillary pruning by losartan results in decreased tissue perfusion and oxygenation in the neonatal mouse heart

**FIGURE 4:**
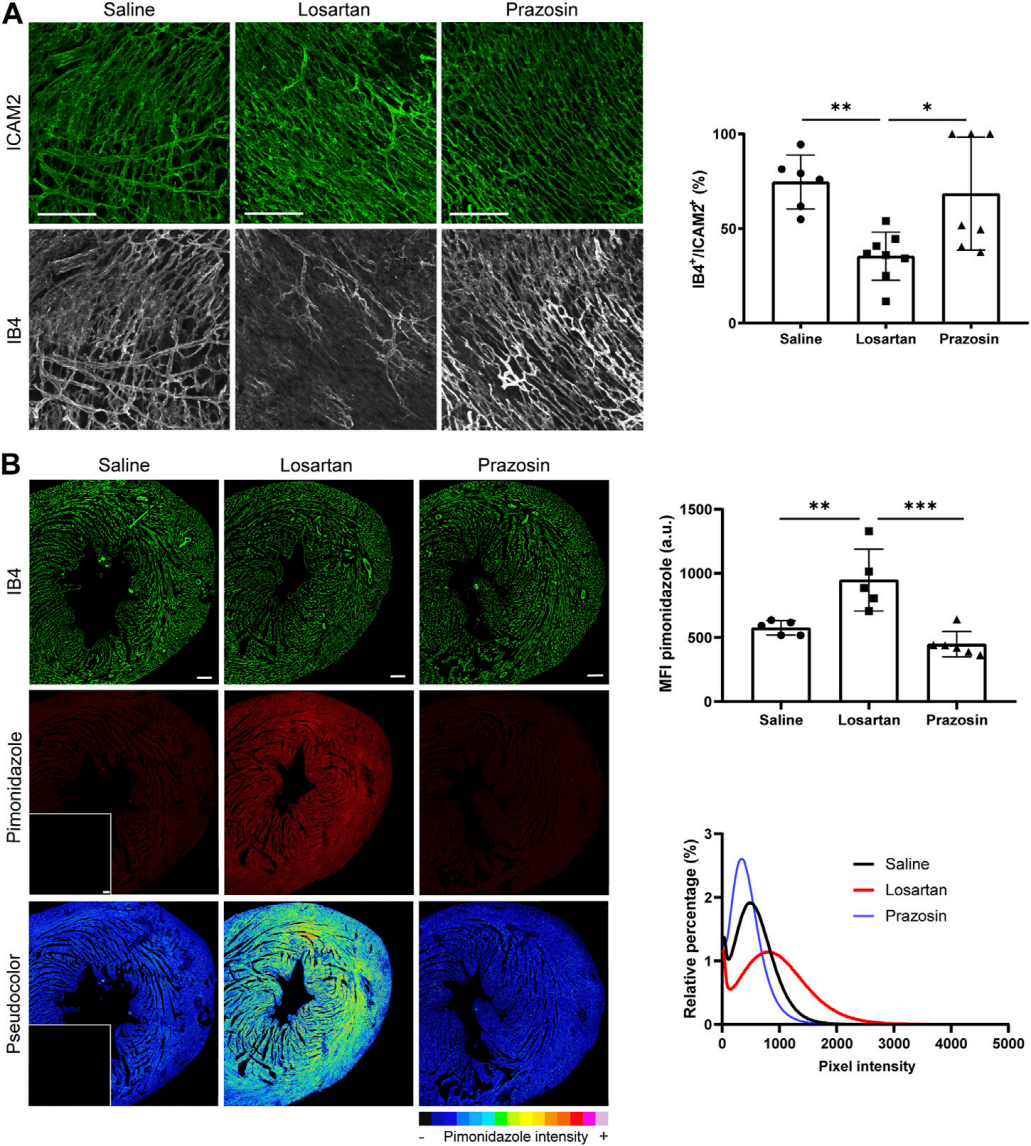
Reduced capillary pruning by the vasodilators losartan or prazosin differentially affects tissue perfusion and oxygenation of the postnatal heart. **(A)**, Confocal microscopy MIP of ICAM2 (green) and IB4 (grey) staining (left) and quantification of tissue perfusion (right) in P7 hearts from mice treated with saline or low doses of losartan or prazosin (10 mg/kg/day) from P2 to P6. Scale bar, 100 µm. **(B)**, Confocal microscopy MIP of vasculature (IB4, green) and hypoxyprobe (red) staining and pseudocolor visualization (left) in the LVFW after conversion to 16-bitmap images in ImageJ and quantification of mean fluorescence intensity (MFI) and relative percentage of hypoxyprobe signal in P7 hearts from mice treated as in A. Insets show negative controls with secondary antibodies only. Scale bar, 100 µm. Bar graphs in A and B show individual values and means ± S.D. and the data were compared by one ANOVA test with Tukey’s multiple comparisons, *p ≤ 0.05, **p ≤ 0.01, ***p ≤ 0.001. See also Supplementary Figures S4, S5.

Although our implemented *ad hoc* algorithm provided capillary diffusion distances (Supplementary Table S1), this parameter can be a good marker of tissue oxygenation only if the vascular networks are perfused with blood flow physiological values (Gkontra et al., 2019), which is unlikely to be the case upon vasodilator treatment (Nehme et al., 2019) (Supplementary Figure S4A). Therefore, we decided to experimentally address cardiac tissue perfusion and oxygenation in hearts treated with low doses of the vasodilators. First, analysis of dextran located outside the blood vessels in thick 3D sections showed that the integrity of the coronary vasculature was preserved in hearts treated with any of the vasodilators (Supplementary Figure S4B). Next, we quantitated tissue perfusion as the percentage of the vasculature positive for i. v. injected isolectin B4 in relation to the total amount of vasculature (ICAM2-positive) in thick 3D images. Interestingly, hearts from losartan-treated mice show a significant reduction in perfusion efficiency, particularly in smaller vessels, compared with the saline-treated control, whereas hearts from prazosin-treated mice showed no mean change in perfusion of the cardiac microvasculature (Figure 4A).

We then assessed cardiac oxygenation by injection of pimonidazole. In the hearts from P7 mice treated with low-dose losartan, there was a significantly higher mean intensity of the hypoxyprobe signal and the frequency histogram was wider and shifted towards higher values compared to the control (Figure 4B), indicating poorer cardiac oxygenation. A pseudo-colour palette also revealed that these hearts had the hypoxyprobe signal regionalized and more heterogeneous, mainly concentrated in the central zone of the myocardium, while the regions adjacent to the great vessels seemed well-oxygenated since they had a very low signal intensity (Figure 4B), suggesting decreased flow distribution to distal territories. Moreover, analysis at P14 of hearts from mice treated with losartan from P2 to P6, still showed higher hypoxyprobe signal compared to saline controls, indicating that the changes induced by the vasodilator at P7 persist over longer time points (Supplementary Figure S5). In contrast, low-dose prazosin-treated hearts showed lower mean intensity of hypoxyprobe signal (Figure 4B), with the frequency histogram narrower and shifted towards values of lower intensity (Figure 4B), suggestive of better oxygenation of these P7 hearts compared to controls and the absence of changes at P14 (Supplementary Figure S5).

### 3.5 Reduced capillary pruning by losartan delays metabolic switch and impairs cardiac function in mouse neonates

We next performed quantitative proteomics using isobaric labeling (TMT) and mass spectrometry to understand at the molecular level the impact of reducing capillary pruning and perturbing tissue oxygenation by treatment with the low dose of the vasodilators. More than 4400 proteins were quantified of which 101 were significantly changed in low dose losartan-treated hearts and 290 in low dose prazosin-treated hearts, and with only 8 significantly increased and 9 decreased proteins common in both treatments (Supplementary Table S2).

**FIGURE 5:**
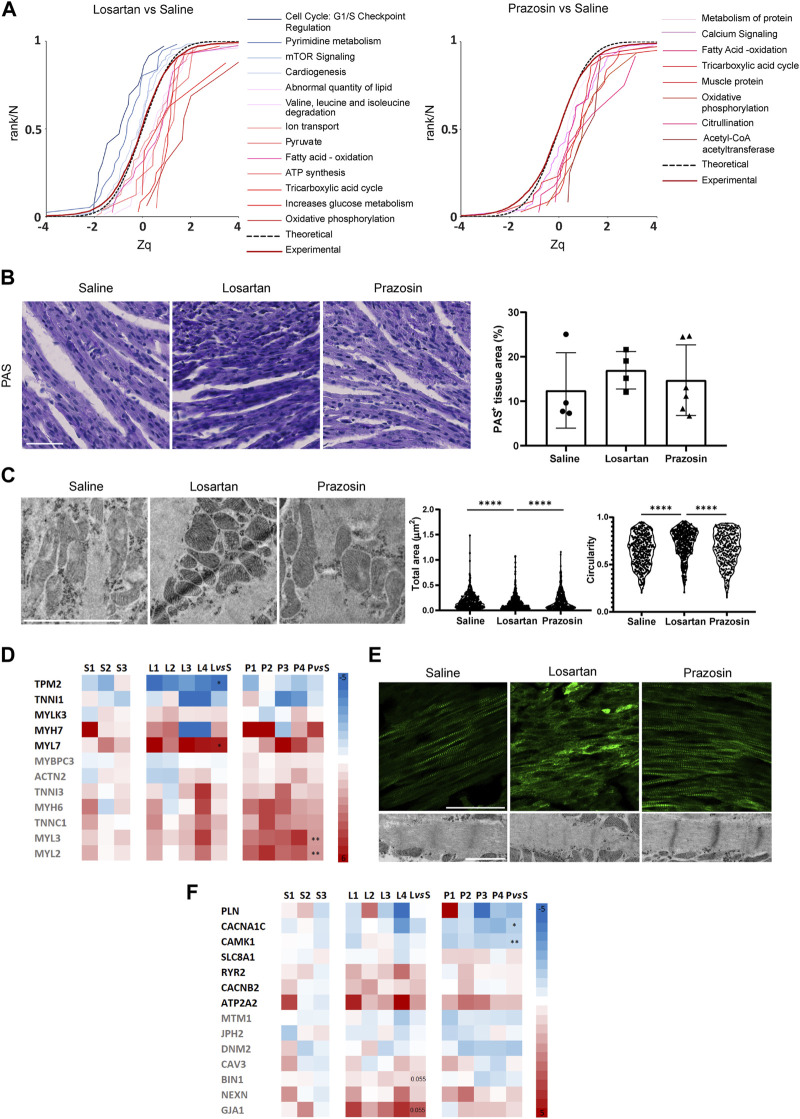
Reduced capillary pruning by the vasodilators losartan or prazosin differentially impacts metabolic switch and maturation of the neonatal heart. **(A)**, Analysis of functional category changes. The graphs are cumulative frequency distributions of standardized log2 protein ratios (Zq), showing significantly decreased (leftward shift of sigmoidal curves) or increased abundance (rightward shift) due to coordinated protein behavior in hearts treated with losartan or prazosin relative to saline controls. The red line represents the null hypothesis (normal distribution) and the dashed line the distribution of all proteins quantified in each experiment. See also Supplementary Table S2. **(B)**, Histochemistry images of PAS staining (left) and quantification of PAS + area (right) in transverse sections of P7 hearts from mice treated with saline or low dose losartan or prazosin from P2 to P6. Scale bar, 50 µm. **(C)**, Transmission electron microscopy (TEM) images (left) and quantification of mitochondria size and circularity (right); *n* = 300 mitochondria from 3 independent mice per condition. Scale bar, 1 µm. **(D)**, Relative abundance of proteins related to immature (black) and mature (grey) sarcomere in losartan or prazosin-treated P7 hearts *versus* saline controls quantitated by Proteomics; the normalized relative abundance values for each vasodilator-treated mouse as well as the mean change *versus* saline (vs*.* S)-treated mice and the statistical significance are shown. **(E)**, Confocal microscopy MIP of cTnn staining (top) and TEM images (bottom) of saline or low dose losartan or prazosin-treated P7 hearts. Scale bar, 50 µm. **(F)**, Relative abundance of proteins related to calcium and conductance (black) and T-tubule development (grey) in losartan or prazosin-treated P7 hearts *versus* saline controls quantitated by Proteomics; the normalized relative abundance values for each vasodilator-treated mouse as well as the mean change *versus* saline (vs*.* S)-treated mice and the statistical significance are shown. Bar graph in B shows individual values and means ± S.D. and the data were compared by one ANOVA test with Tukey’s multiple comparisons. Graphs in C show single cell values and were compared by Kruskal-Wallis test with multiple comparisons corrected by Dunn’s method, ****p ≤ 0.0001. See also Supplementary Figure S6.

A metabolic switch from anaerobic glycolysis, the main source of energy in the first days, to oxidative phosphorylation (OXPHOS), mainly fatty acid oxidation (FAO), occurs in mouse cardiomyocytes during the first weeks after birth (Lopaschuk and Jaswal, 2010). Analysis of changes in functional categories due to coordinated protein behavior using the SBT model (Garcia-Marques et al., 2016) showed that both vasodilators significantly increased ATP synthesis, OXPHOS, FAO and tricarboxylic acid cycle categories compared to controls (Supplementary Table S2). However, while losartan treatment significantly increased glucose metabolism, pyruvate and abnormal lipid quantity categories, suggesting persistent glycolysis and inefficient FAO, also supported by increased branched amino acid degradation, prazosin treatment significantly increased acetyl CoA-acetyl transferase, an essential enzyme in FAO (Figure 5A; Supplementary Table S2), suggesting that the two vasodilators differentially influenced the expected postnatal metabolic change in neonatal hearts. We then performed histochemistry with periodic acid Schiff (PAS) to directly assess glycogen abundance as a surrogate marker of glycolytic flux (Fajardo et al., 2021; Santamans et al., 2021). PAS staining revealed that glycogen tended to be more abundant in hearts treated with losartan, but not prazosin, compared to saline controls (Figure 5B). Since metabolic switch in postnatal cardiomyocytes is also accompanied by increased mitochondrial biogenesis and fusion (Guo and Pu, 2020), we analyzed hearts by transmission electron microscopy (TEM) which revealed smaller, rounder mitochondria in losartan-treated hearts compared with saline controls and in contrast to prazosin (Figure 5C). These data support that reduced capillary pruning by losartan and subsequent tissue hypoxia contribute to physiological metabolic switch in postnatal cardiomyocytes (Menendez-Montes et al., 2016). In addition, losartan significantly reduced mTOR signalling and pyrimidine metabolism, while prazosin significantly increased citrullination and protein metabolism, suggesting lower and higher anabolism, respectively, as also supported by the increase in muscle protein category in prazosin-treated hearts (Figure 5A; Supplementary Table S2).

Postnatal cardiomyocyte metabolic switch is also coupled to changes in the abundance and isoforms of several contractile proteins along with myofibril expansion (Guo and Pu, 2020). Accordingly, losartan treatment resulted in significantly increased abundance of the immature sarcomere protein myosin light chain 7 (MYL7), higher levels of the β isoform of myosin heavy chain/myosin heavy chain 7 (MYH7) and significantly lower expression of tropomyosin 2 (TPM2) while the hearts treated with low-dose prazosin contained significantly higher abundance of isoforms and proteins related to sarcomere maturation (Yin et al., 2015; Guo and Pu, 2020; Ahmed et al., 2022), such as myosin light chain 2 (MYL2) and myosin light chain 3 (MYL3), and also higher levels of cardiac troponin isoform C1 (TNNC1), the α isoform of myosin heavy chain/myosin heavy chain 6 (MYH6), cardiac troponin isoform I3 (TNNI3), actinin 2 (ACTN2) and myosin binding protein C3 (MYBPC3) (Figure 5D). Indeed, confocal microscopy of cardiac troponin (cTnn)-stained sections and TEM confirmed better organized sarcomeres with Z-bands and patent M-lines in prazosin-treated hearts, compared to a more disorganized cTnn and sarcomeres in losartan-treated hearts and an intermediate phenotype in saline controls (Figure 5E). These results suggest accelerated cardiomyocyte maturation in the case of prazosin, whereas this process appeared to be delayed with losartan treatment, probably related to its differential impact on tissue oxygenation.

Electrical conductance maturation also involves changes in the abundance of calcium-related channels and proteins and their relocation to the sarcoplasmic reticulum (Guo and Pu, 2020; Karbassi et al., 2020). The ion transport and the calcium signaling categories were found significantly increased in losartan- and prazosin-treated hearts, respectively (Figure 5A; Supplementary Table S2). Moreover, there were variations in the expression of proteins related to T-tubule development, such as gap junction protein α1/connexin-43 (GJA1) and bridging integrator 1/amphiphysin II (BIN1), which were increased in losartan-treated hearts, and to calcium and conductance, such as calcium voltage-gated channel subunit α1/cav1.2 (CACNAC1) and calcium/calmodulin-dependent protein kinase I (CAMK1), which were significantly decreased in prazosin-treated hearts (Figure 5F; Supplementary Table S2). Together these data suggest an additional impact of the vasodilators on electrical conductance.

We next investigated whether the observed changes in contractility and conductance-related categories and proteins had any functional impact on cardiac function by performing echography in neonates at P7, after saline or vasodilator administration, and at P1, before any treatment (Supplementary Table S3). Low doses of losartan and prazosin tended to reduce the increment in left ventricular mass at P7 (Supplementary Figure S5A), consistent with the observed decrease in heart weight (Supplementary Figures S2B, S3B). Interestingly, left ventricular posterior wall deformation, used clinically as a marker of contractility (Voigt and Flachskampf, 2004), increased at P7 *versus* P1 throughout the maturation of control hearts but appeared to decrease in hearts treated with losartan or prazosin (Supplementary Figure S6A). This may indicate abnormal left ventricular wall contractility. Indeed, similar results were observed with the canonical contractility parameters ejection fraction and fractional shortening, further supporting defective contractility in hearts treated with the vasodilators (Supplementary Figure S6A). Cardiomyocyte maturation also implies adequate electrical coupling for efficient conductivity. Electrocardiogram analysis at P7 (after treatment) showed a mild acceleration of heart rate in mice treated with losartan or prazosin (Supplementary Figure S6B; Supplementary Table S3), as expected after vasodilator administration, and no significant changes in atrial conductance (P wave, PR interval) but a significantly shorter normalized QRS complex (Supplementary Figure S6B; Supplementary Table S3). Given that the *in vitro* effects reported for losartan and prazosin on metabolism, mitochondria, contractility or conductance of cardiomyocytes (Luo et al., 2001; Xu et al., 2009; Pennanen et al., 2014; Gerena et al., 2017) do not fully align with those we observed *in vivo*, our data suggest that treatment of neonatal mice with losartan or prazosin results in defective cardiac ventricular contractility and accelerated electrical conductivity, at least in part by affecting capillary pruning and cardiomyocyte maturation.

### 3.6 Modulation of capillary pruning by vasodilator treatment affects cardiomyocyte cell cycle exit in the postnatal heart

**FIGURE 6:**
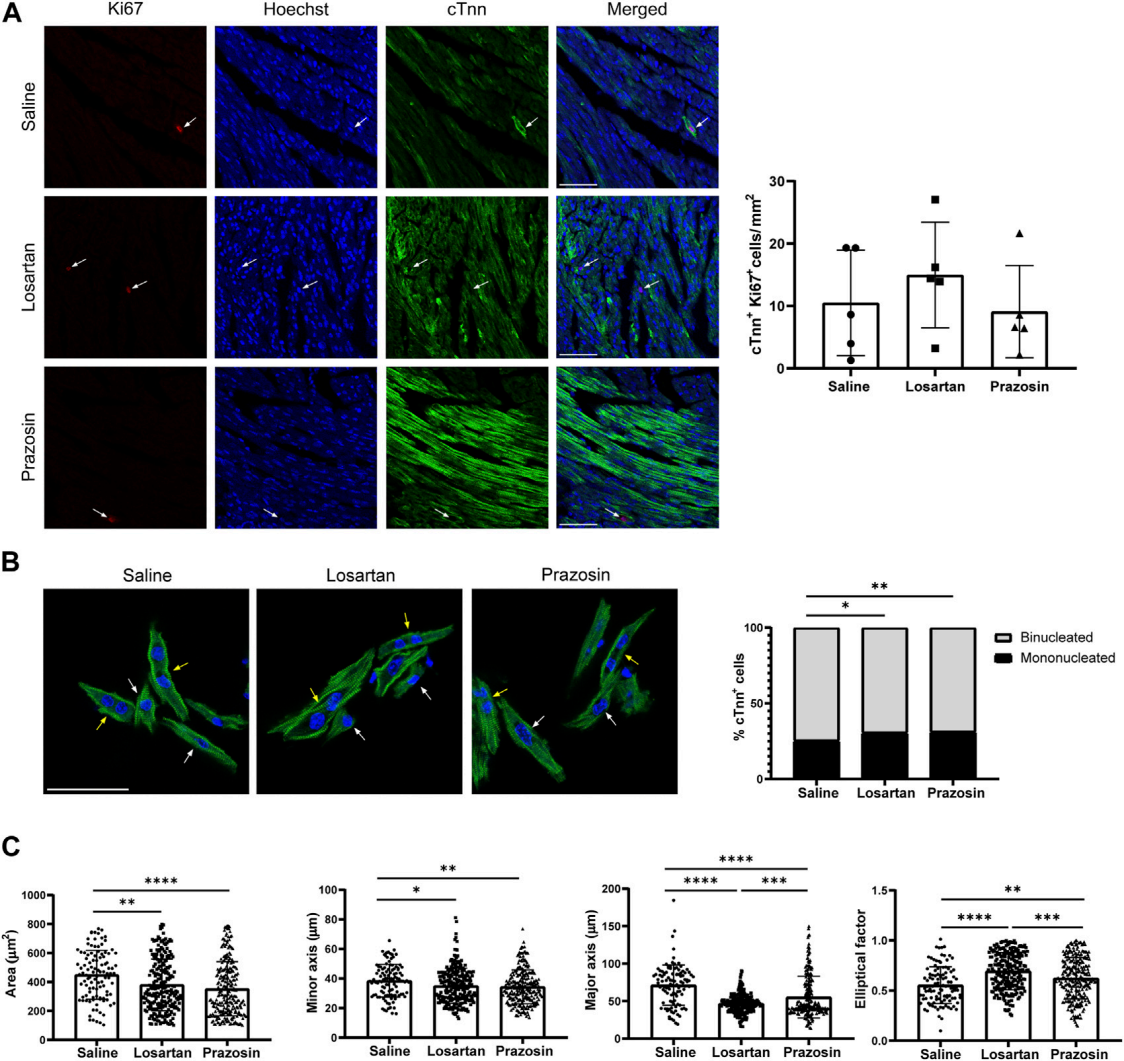
Reduced capillary pruning by losartan or prazosin treatment affects cardiomyocyte cell cycle, size and morphology. **(A)**, Confocal microscopy MIP of Ki-67 (red), Hoechst (blue) and cTnn (green) staining (left) and quantification of proliferating cardiomyocytes (Ki-67+/cTnn+) in P7 hearts from mice treated with saline or low dose losartan or prazosin from P2 to P6. Arrows indicate Ki-67+ cardiomyocyte nuclei. Scale bar, 50 µm. **(B)**, Confocal microscopy images of cTnn (green) and DAPI (blue) staining in cardiomyocytes isolated from fixed P7 hearts (left) and quantification of the percentage of mono and binucleated cardiomyocytes in hearts from mice treated with saline or low dose losartan or prazosin. Data from *n* = 1392 cells (saline, from 7 hearts), *n* = 1115 cells (losartan, from 5 hearts) and *n* = 1628 cells (prazosin, from 5 hearts). White and yellow arrows indicate mononucleated and binucleated cardiomyocytes, respectively. Scale bar, 50 µm. **(C)**, Quantification of the area, minor and major axis, and elliptical factor in cardiomyocytes isolated from hearts of mice treated as in B. Data from *n* = 105 cells (saline), *n* = 229 cells (losartan) and *n* = 261 cells (prazosin). Bar graphs in A and C shows individual values from mice or cells, means ± S.D. and the data were compared by one ANOVA test with Tukey’s multiple comparisons. Graph in B shows percentage distribution and data were analysed with Fisher exact test with Bonferroni-Dunn *p*-value adjustment for multiple comparisons, *p ≤ 0.05, **p ≤ 0.01, ***p ≤ 0.001, ****p ≤ 0.0001.

Another feature of postnatal cardiomyocyte maturation, which correlates with the metabolic switch, is cell cycle exit and cell division arrest, resulting in a transition from immature, mononucleated and proliferative cardiomyocytes to mature, binucleated and non-proliferative cardiomyocytes during the first weeks after birth in mice (Guo and Pu, 2020). In the proteomics analysis, the category of cell cycle (G1/S checkpoint regulation) was significantly reduced only in losartan-treated hearts (Figure 5A; Supplementary Table S2). Furthermore, immunostaining of paraffin-embedded cardiac sections of P7 mice with anti-Ki-67 (a marker of cells in G1/G2/M phase) and cTnn showed that losartan, but not prazosin, appeared to increase the abundance of proliferating cardiomyocytes (cTnn + Ki-67+) (Figure 6A). We then isolated cardiomyocytes from P7 hearts of losartan-treated mice and observed a significantly higher proportion of mononucleated *versus* binucleated cardiomyocytes and accordingly, these cardiomyocytes were smaller and rounder compared with controls (Figures 6B, C), consistent with a delayed cardiomyocyte cell division arrest (Figure 5A; Supplementary Table S2). Unexpectedly, cardiomyocytes isolated from prazosin-treated hearts also contained a higher proportion of mononucleated cells and were smaller and less elongated than controls (Figure 6B), which, without an increase in Ki-67+ cardiomyocytes (Figure 6A), would suggest a premature and altered exit from the cell cycle in parallel with accelerated maturation of the contractile machinery (Figures 5C, D).

Taken together, these findings demonstrate that reduced capillary pruning, by losartan or prazosin, affects cardiomyocyte maturation, but in different ways. Losartan treatment with concomitant tissue hypoxia and delayed metabolic switch prevents cell cycle arrest and proper cardiomyocyte maturation and growth while treatment with prazosin, that does not impair tissue oxygenation or the metabolic switch, seems to lead to premature cardiomyocyte maturation.

## 4 Discussion

Capillary pruning had previously been characterized in developing vascular plexus with a simplified geometry such as the chick chorioallantoic membrane, embryonic zebrafish and postnatal mouse retina (Santamaria et al., 2020). One of the main novelties of our work is the implementation of a method for the visualization and quantification of capillary pruning events in complex tissues by Imaris^®^ reconstruction of 3D volumes stained for markers that identify “empty sleeves”, the hallmark of segment pruning. Interestingly, similar 3D reconstruction has recently been used to detect the presence of capillary pruning events in the adult mouse cerebral vasculature (Gao et al., 2020), pointing to the robustness of this approach in different vascular plexuses. This tool allowed us to recognize and characterize capillary pruning in the postnatal mouse heart for the first time. This remodeling process takes place already at P1, reaches its peak at P7 and lasts at least until P14 (assessment at later stages is technically difficult due to the higher density and complexity of the network), finally resulting in a well-patterned network at P14 that we show to be more efficient for oxygen delivery than at P7. Furthermore, the presence of areas of higher endothelial cell density in P7 points to intravascular endothelial cell rearrangements as the mechanism underlying pruning in the postnatal coronary network, similar to postnatal mouse retina and zebrafish embryonic brain (Chen et al., 2012; Franco et al., 2015) and in contrast to mammalian adult brain vessels in which regression appears to be related to occlusion (Gao et al., 2020; Schager and Brown, 2020). However, given the limitations of the cell density mapping tool, especially in 3D volumes, confirmation would require complementary approaches to assess endothelial cell polarization, such as visualization of the nucleus/Golgi axis (Pena et al., 2021) by immunostaining or in the GNRep reporter mouse model (Barbacena et al., 2019).

Based on previous reports showing that modulation of blood flow by vasodilation (with losartan or captopril) or vasoconstriction (with angiotensin II) had an impact on the rate of capillary pruning in models of pathophysiological angiogenesis in the retina (Lobov et al., 2011; Franco et al., 2016), we showed that treatment with two different vasodilators, losartan (ATR1 inhibitor) and prazosin (α1R antagonist) reduced pruning in the postnatal retina, but also in the postnatal coronary vasculature. Both vasodilators, and mainly at low doses, seemed to increased blood flow sensing in the overall vasculature while decreasing it in large SMA + vessels, as we showed in the blood flow-sensitive Klf2-GFP reporter mouse model (Weinreich et al., 2009). In a recent report, Klf4 has similarly been used to study the impact of captopril and angiotensin II on the postnatal retina microvasculature (Barbacena et al., 2022). Given the reduced perfusion in small vessel induced by losartan in contrast to prazosin, Klf2 expression may be responding to different signals in capillary endothelial cells from mouse hearts treated with losartan (pulsatile/oscillatory blood flow (Heckel et al., 2015)) and prazosin (laminar flow (Dekker et al., 2002)).

Using our previously implemented *ad hoc* algorithm for segmentation and analysis of 3D microvascular networks (Gkontra et al., 2018), we observed that low-dose losartan impacted mostly on the diameter of vessels and that the reduction in the abundance of pruning events correlated with the number of segments and bifurcations similarly to controls, resulting in a slight expansion of microvascular density and decreased network heterogeneity. Unexpectedly for the mild average effects on the network, it also resulted in a patently heterogeneous myocardial perfusion and oxygenation, alternating well-perfused and oxygenated areas around the large vessels with poorly perfused and oxygenated areas in the rest of the capillary-perfused tissue. This is in line with previous computational simulations in which reducing pruning in the postnatal retina network had a negative impact in the distribution of blood flow from the large vessels to the distal capillaries promoting heterogeneity in tissue oxygenation (Watson et al., 2012). Reduced perfusion suggests that blood stagnation occurs in losartan-treated hearts which, associated with possible plasma skimming effects, could further contribute to heterogeneity of blood flow and oxygen distribution (Roy and Secomb, 2021) and even further decrease in pruning, given the suggested role of red blood cells in the process (Zhou et al., 2021). Our observations of larger retinal vein diameter (not shown) and increased abundance of 5–8 µm vessels in the heart also suggest that reducing pruning in the case of losartan may prevent the elimination or favor the formation of arterial-venous shunts that would increase blood flow heterogeneity and aggravate oxygenation of distal territories as observed in arteriovenous malformations or in the tumor vasculature (Pries et al., 2010; Bernabeu et al., 2020; Park et al., 2021). It will be interesting to perform blood flow simulations and complementary approaches in the vascular network of losartan-treated hearts to confirm this point. Our data also highlight the need of actual measurement of blood flow and/or tissue perfusion in developmental and pathological networks, given the observed discrepancy between structure (slightly impaired) and function (patent decreased perfusion) in losartan-treated hearts, but also given the lack of robustness of theoretical estimation of tissue perfusion/oxygenation based on network structural characteristics when assuming physiological blood flow and pressure drop which may be confounding factors as we previously reported in the pig infarcted heart model (Gkontra et al., 2019).

Conversely to losartan, treatment with low-dose prazosin not only preserved perfusion, but even improved tissue oxygenation, suggesting additional network effects. As shown by the 3D image analysis of the microvasculature this distinct impact may rely on the concurrent expansion of the microvasculature induced by prazosin, driving blood flow to the distal territories and avoiding hypoxia. Prazosin is known to promote capillary splitting in the skeletal muscle in a nitric oxide-dependent manner (Williams et al., 2006a). The presence of increased number of segments, bi/trifurcations, node complexity, etc. in prazosin-treated hearts strongly suggest the enhancement of capillary splitting, known to contribute to the expansion of the coronary microvasculature during the first weeks after birth (van Groningen et al., 1991). The early stages of postnatal development of the heart could serve as a model of coexisting capillary pruning and splitting and their possible links during microvascular remodeling (Santamaria et al., 2020). Proposing the reduction of pruning with losartan or with captopril to increase microvascular density in pathologies as myocardial infarction or oxygen-induced retinopathy (Barbacena et al., 2022) would likely be accompanied by the formation of arterial-venous shunts that negatively impact blood flow distribution unless a parallel expansion of capillaries is achieved as observed with prazosin. These different effects of the vasodilators appear to be related to the preferential expression of ATR1 *versus* α1AR receptors in small resistance vessels (Chai et al., 2010) and large arterioles (Faber, 1988), respectively, rather than to their different vasodilator power, since losartan and prazosin, at doses similar to those used in our study, caused equivalent decreases in blood pressure at least in adult mice or rats (Makaritsis et al., 1998; Kristek et al., 2013). 3D approaches in the postnatal coronary microvasculature will also help to understand the real role of the magnitude and gradients of blood flow and shear stress in promoting segment pruning and maturation of real (not idealized) networks (Gkontra et al., 2019; Alberding and Secomb, 2021).

Our study demonstrates experimentally that adequate remodeling by capillary pruning is necessary for an efficient vascular network in the postnatal heart, consistent with a recent report using intravital microscopy in neonatal mouse skin (Kam et al., 2023), since this had previously only been analyzed by computational simulations of postnatal mouse retinal plexuses (Watson et al., 2012). Although our study focused on the analysis of capillary pruning in developing hearts and not in pathological conditions, it is tempting to speculate that after myocardial infarction in which blood flow and microvasculature architecture change, this pruning remodeling may occur and explain, at least in part, the capillary reduction observed a few days after myocardial infarction (Gkontra et al., 2018). In the same line, in brain, recent reports indicate that capillary pruning is associated with aging, pathology, and loss of neuronal and cognitive activity (Gao et al., 2020). Therefore, our results posit modulation of capillary pruning by vasodilation as a valuable strategy in cardiac ischemia, as we already predicted in the infarcted pig heart model (Gkontra et al., 2019), but maybe also in Alzheimer’s disease and amyotrophic lateral sclerosis (Religa et al., 2013; Crivello et al., 2019). How to apply this knowledge in diseases in which defective capillary pruning leads to aberrant and inefficient vascular networks, such as in arteriovenous malformations (Corti et al., 2011) or in tumors (Bernabeu et al., 2020) deserves further investigation. In all cases the fine regulation and actions that blood flow has on this process will need to be taken into account, since, as our data show, imprecise modulation can lead to tissue hypoxia.

Our data also demonstrate that pruning appears to be necessary for the microvasculature to ensure perfusion-metabolism matching and cardiac function (Roy and Secomb, 2021). Decreased pruning (by low doses of losartan) results in heterogeneous tissue perfusion and oxygenation that directly impacts cardiomyocyte metabolism by preventing the normal metabolic transition from glycolysis to fatty acid oxidation and interfering with cardiomyocyte maturation (Guo and Pu, 2020), in particular the exit from the cell cycle, resulting in small, proliferative, immature cardiomyocytes and impaired cardiac contractility and conductance. Of interest, losartan-treated hearts showed reduced pyrimidine metabolism and changes in aminoacid metabolism consistent with those captured by metabolomics and proteomics (but in the opposite direction) in a mirror model of increased segment pruning in the brain vasculature (Gao et al., 2020). This strongly supports that adequate pruning is required for proper oxygenation and metabolism in different tissues (heart and brain) and at different developmental stages (neonate and adult). Conversely, reduced pruning coupled with additional expansion of the microvasculature, as in prazosin treatment, appears to drive early cardiomyocyte maturation, evidenced by increased abundance of adult contractile and sarcomere protein isoforms and likely related to increased tissue perfusion and oxygen levels, known to induce cardiomyocyte maturation (Guo and Pu, 2020). Nevertheless, precocious cardiomyocyte maturation in developing and postnatal heart through interference with cytoskeleton or metabolism (Sun et al., 2019; Gan et al., 2022), led to reduced myocardial growth and compaction, in line with the abnormal cardiac contractility and conductance observed in prazosin-treated neonatal mice. However, prazosin treatment may still be beneficial for certain ischemic heart disorders in adult mice.

Metabolic plasticity is a hallmark of cardiomyocytes that allow them to adapt to changing needs for example after birth but also in stressful conditions such as lack of hypoxia sensing machinery (Menendez-Montes et al., 2021) or stress-response kinases (Santamans et al., 2021) but this capacity decreases in the adult hearts in which reduced metabolic adaptability upon damage can end up in heart failure (Karwi et al., 2018). Our findings highlight this plasticity of cardiac metabolism, especially in neonates, and open up possibilities for interventions based on modulation of microvasculature remodeling in addition to those previously demonstrated by nutritional [malonate, (Bae et al., 2021); fatty acids (Cao et al., 2019); amino acids (Menendez-Montes et al., 2021)] or genetic manipulations (Magadum et al., 2020; Santamans et al., 2021). Future studies are necessary to investigate the effects of modulating pruning on metabolic adaptations after cardiac ischemia.

Our study opens new avenues for judicious manipulation of blood flow gradients with vasodilators to modulate pruning and improve tissue perfusion in certain pathophysiological contexts. However, given that vasodilators can have other systemic cardiovascular effects, identifying the molecular players involved in capillary pruning in the heart would allow cell type-specific and time-defined manipulations of the process. Our endothelial cell density mapping tool suggests underlying mechanisms compatible with the current model of endothelial migration towards the high-flow vessel (Barbacena et al., 2016). Thus, actors similar to those described in pruning in the postnatal retina model or in the zebrafish brain (e.g. Rac1, whose levels appear decreased in our proteomic analysis of hearts treated with losartan or prazosin, Supplementary Table S2) could contribute to the remodeling of the coronary microvasculature despite the different patterns of blood flow in these territories (pulsatile and cyclic in the heart). Among them, endoglin, Alk1, CDS2 and Rac1 genes have been found associated with congestive heart failure (GDA; https://www.disgenet.org/). It would be interesting to analyze the contribution of these genes to capillary pruning in the heart and to dissect whether the pathogenesis of congestive heart failure may be related, at least in part, to alterations in postnatal coronary microvascular network remodeling and thus cardiomyocyte metabolism and maturation. In summary, our study serves as a first proof of concept to demonstrate the existence of capillary pruning and its relevance in coupling perfusion and metabolism with postnatal heart maturation and function.

## Data Availability

The original contributions presented in the study are included in the article/Supplementary Materials, further inquiries can be directed to the corresponding author.

## Supplementary material

SUPPLEMENTARY TABLE S2 Quantitative proteomics of P7 neonatal hearts treated with saline, losartan or prazosin. Lists of the quantified proteins with their relative abundance and statistical significance and of the selected significantly changed categories with their corresponding proteins depicted in Figure 5A according to proteomic analysis in P7 mouse hearts treated with saline or 10 mg/kg losartan or prazosin.

SUPPLEMENTARY MOVIE S1 3D reconstruction of the microvasculature of a neonatal heart from a saline-treated P7 mouse. Confocal microscopy images were acquired by mosaic scanning of the whole heart stained with ICAM2 (green) with sections every 2 µm and used for three-dimensional reconstruction with Imaris®.

SUPPLEMENTARY MOVIE S2 3D reconstruction of the microvasculature of a neonatal heart from a low dose losartan-treated P7 mouse. Confocal microscopy images were acquired by mosaic scanning of the whole heart stained with ICAM2 (green) with sections every 2 µm and used for three-dimensional reconstruction with Imaris®.

SUPPLEMENTARY MOVIE S3 3D reconstruction of the microvasculature of a neonatal heart from a high dose losartan-treated P7 mouse. Confocal microscopy images were acquired by mosaic scanning of the whole heart stained with ICAM2 (green) with sections every 2 µm and used for three-dimensional reconstruction with Imaris®.

SUPPLEMENTARY MOVIE S4 3D reconstruction of the microvasculature of a neonatal heart from a low dose prazosin-treated P7 mouse. Confocal microscopy images were acquired by mosaic scanning of the whole heart stained with ICAM2 (green) with sections every 2 µm and used for three-dimensional reconstruction with Imaris®.

SUPPLEMENTARY MOVIE S5 3D reconstruction of the microvasculature of a neonatal heart from a high dose prazosin-treated P7 mouse. Confocal microscopy images were acquired by mosaic scanning of the whole heart stained with ICAM2 (green) with sections every 2 µm and used for three-dimensional reconstruction with Imaris®.
