# Supplementary material for: Capillary pruning couples tissue perfusion and oxygenation with cardiomyocyte maturation in the postnatal mouse heart
Source: Front Cell Dev Biol. 2023 Nov 7;11:1256127. doi: 10.3389/fcell.2023.1256127 (PMC10661946; doi:10.3389/fcell.2023.1256127)
Supplement: Supplementary file 2 [file DataSheet1.PDF]

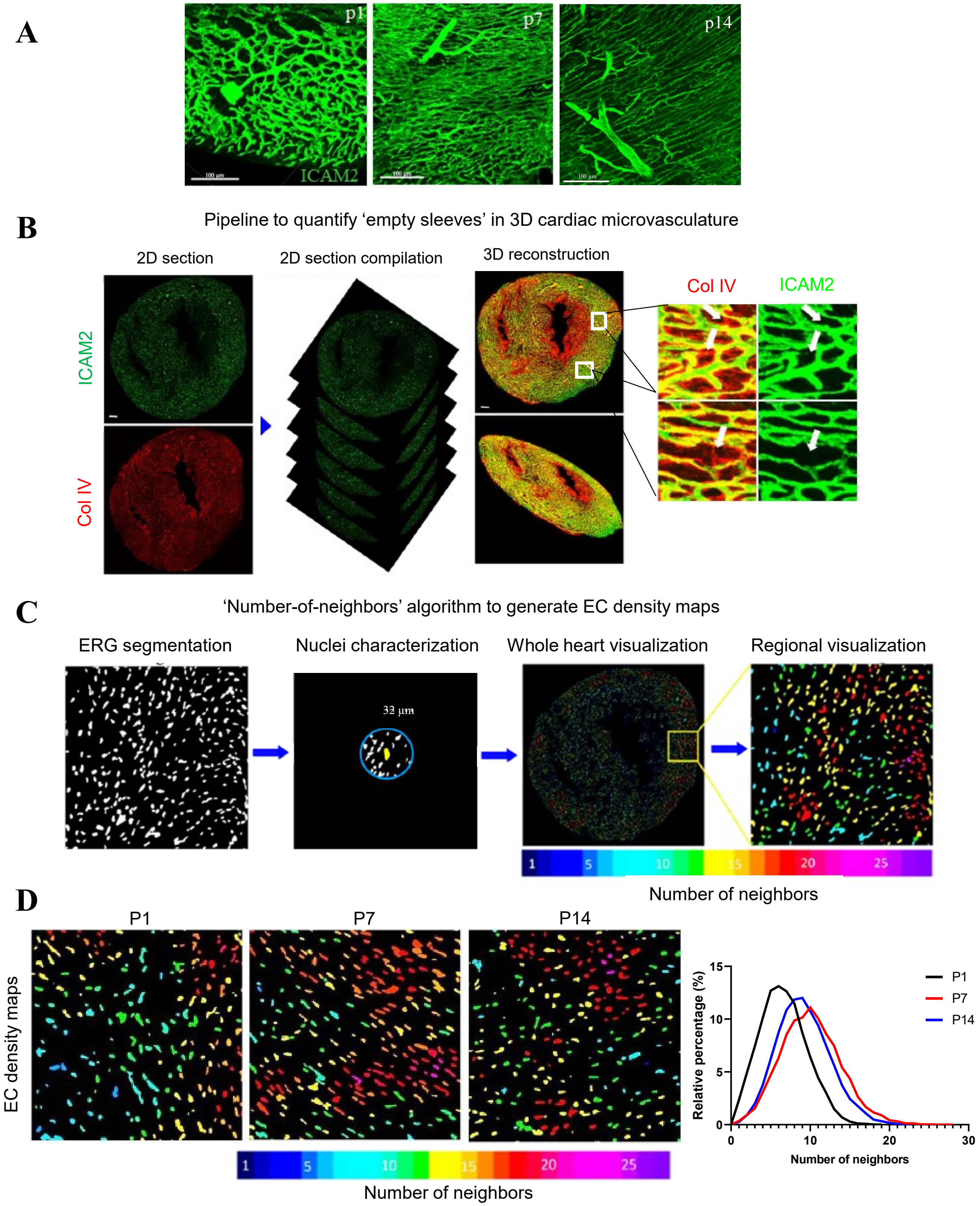

Figure S1. Imaging analysis of the remodeling of the mouse cardiac microvasculature during the first weeks after birth (related to Figure 1). A, ICAM2-based 3D rendered images of neonatal hearts from P1 to P14. Scale bar 100 μm. **B**, Pipeline for acquisition, reconstruction and quantification of pruning events in 3D images of the microvasculature; arrows indicate 'empty sleeves' (collagen IV positive/ICAM2 negative). **C**, Pipeline of the implementation of an image-based analysis tool to map regional endothelial cell density as a function of 'number of neighbors' counted in a 32 μm radius around each nucleus (yellow). **D**, Endothelial cell density maps in P1, P7 and P14 mouse hearts and corresponding relative percentage histograms of the number of neighbors per endothelial cell nucleus.

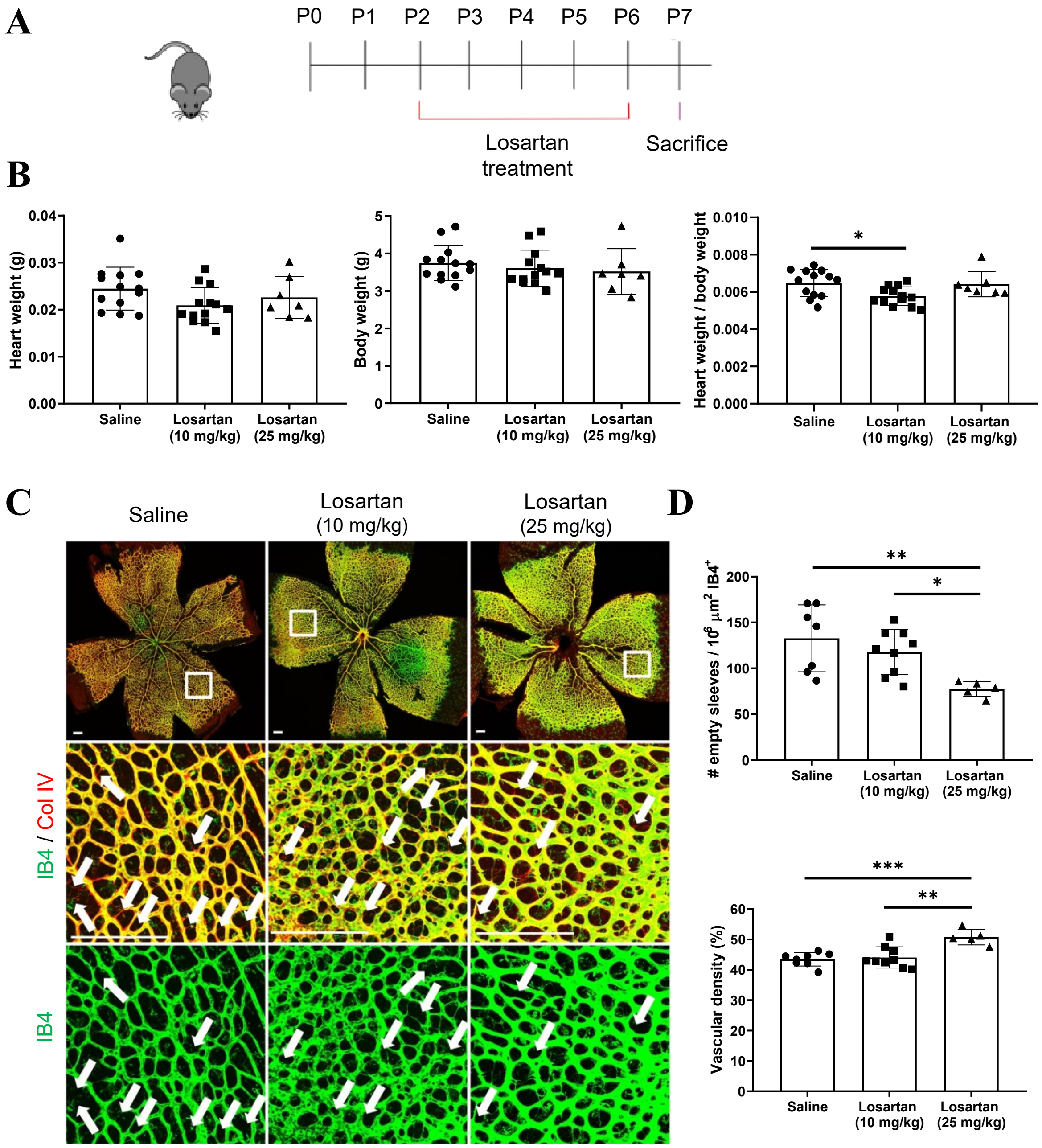

Figure S2 (related to Figure 2). Dose-response effects of losartan in neonatal P7 mice and their retinal capillary pruning. A, Schematic of protocol for daily administration of saline or losartan (at doses of 10 or 25 mg/kg) subcutaneously from P2 to P6 and sacrifice at P7. **B**, Quantification of heart and body weight, and their ratio, in P7 mice treated as in A. **C**, Confocal microscopy MIP of IB4 (green) and Col IV (red) staining in P7 retinas of mice treated as in A. Arrows indicate 'empty sleeves'. Scale bars, 200 μm **D**, Quantification of the number of empty sleeves and vascular density in P7 retinas of mice treated as in A. Data in B, C and D were compared by one-way ANOVA with Tukey's post-hoc corrections for multiple testing, *p≤0.05, **p≤0.01, ***p≤0.001.

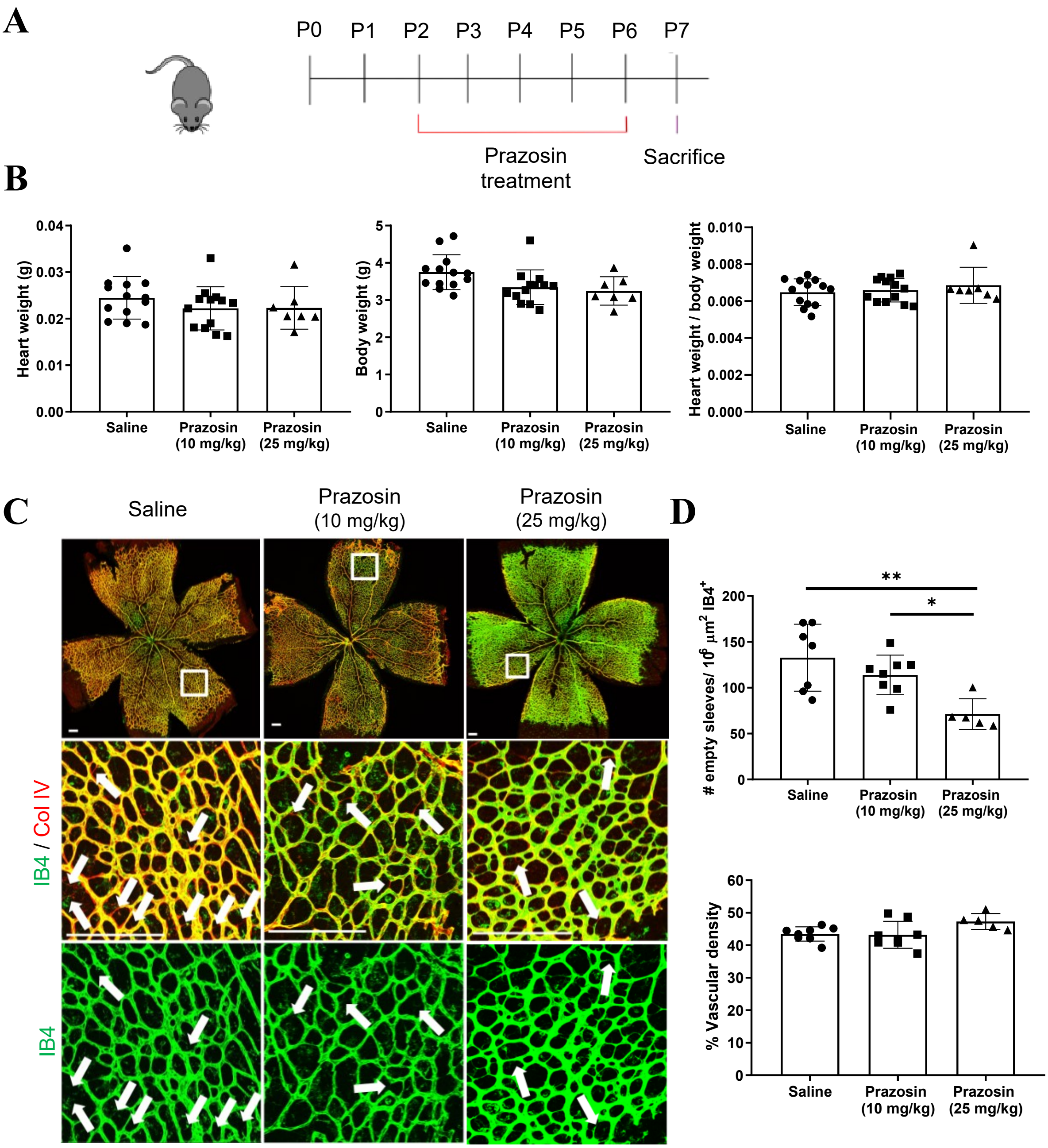

Figure S3 (related to Figure 3). Dose-response effects of prazosin in neonatal P7 mice and their retinal capillary pruning. A, Schematic of protocol for daily administration of saline or prazosin (at doses of 10 or 25 mg/kg) subcutaneously from P2 to P6 and sacrifice at P7. **B**, Quantification of heart and body weight, and their ratio, in P7 mice treated as in A. **C**, Confocal microscopy MIP of IB4 (green) and Col IV (red) staining in P7 retinas of mice treated as in A. Arrows indicate 'empty sleeves'. Scale bars, 200 μm **D**, Quantification of the number of empty sleeves and vascular density in P7 retinas of mice treated as in A. Data in B, C and D were compared by one-way ANOVA with Tukey's post-hoc corrections for multiple testing, $*p \leq 0.05$, $**p \leq 0.01$. The images and values for the saline-treated control mice are the same as in Figure S3, as the experiments were performed in parallel, but these data are presented separately for clarity.

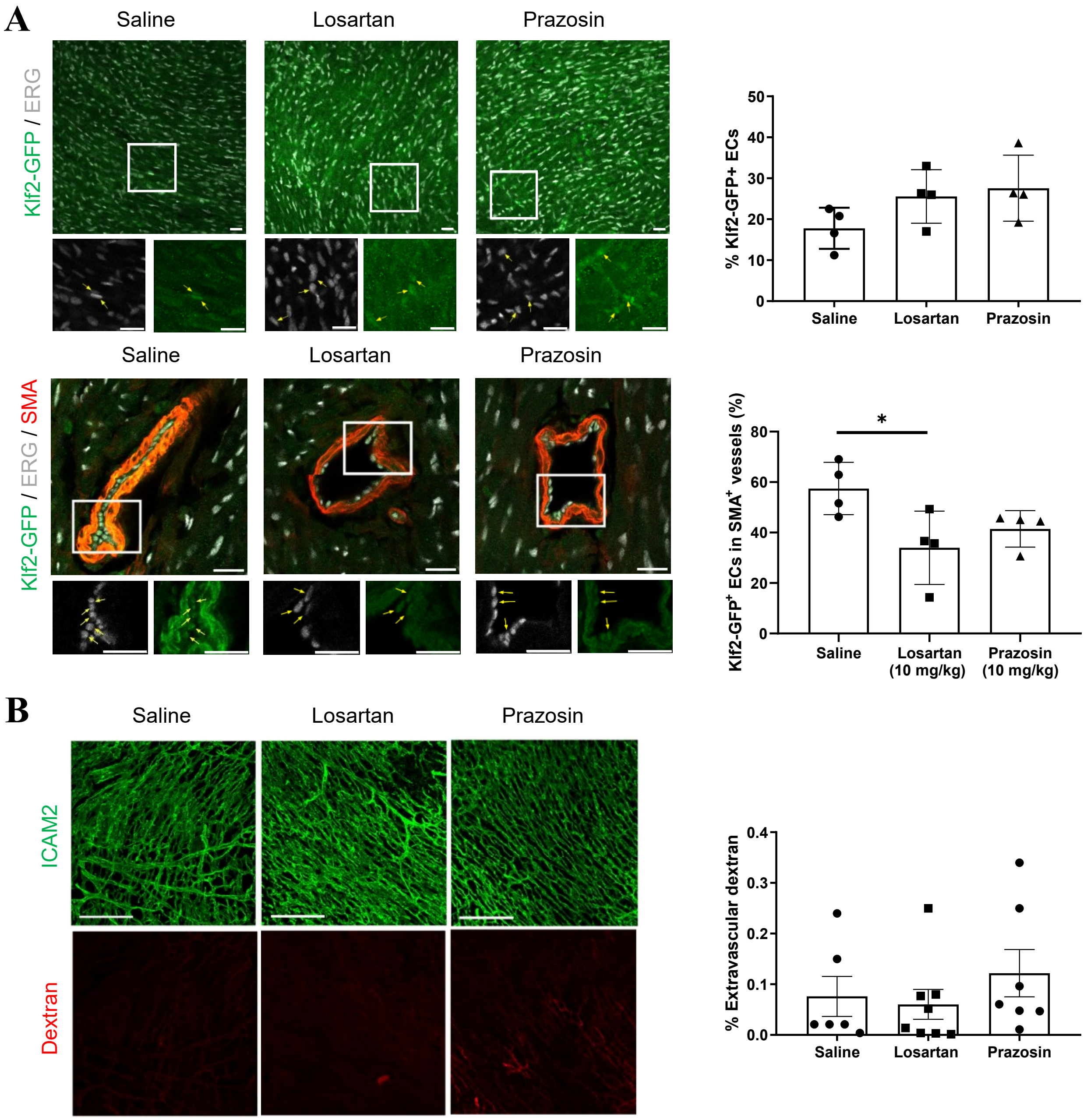

Figure S4 (related to Figure 4). Effect of the vasodilators losartan and prazosin on blood flow and vascular integrity. A, Confocal microscopy MIP of Klf2-GFP (green) and Erg (grey) (top) and Klf2-GFP (green), SMA (red) and Erg (grey) (bottom) in P7 hearts of mice treated with saline or low dose losartan or prazosin from P2 to P6 (left) and quantification of % Klf2-GFP+ endothelial cells in total vasculature or in SMA+ large vessels (right). Scale bars, 25 µm. Arrows at magnifications indicate double positive nuclei (GFP/Erg). **B**, Confocal microscopy MIP of ICAM2 (green) and i.v. injected dextran (red) in P7 hearts from mice treated with saline or low doses of losartan or prazosin (left) and quantification of extravascular dextran signal (right). ICAM2 images are the same as those used for perfusion analysis. Scale bars, 100 microns. Data in A and B were compared by one-way ANOVA with Tukey's post-hoc corrections for multiple testing, *$p \leq 0.05$.

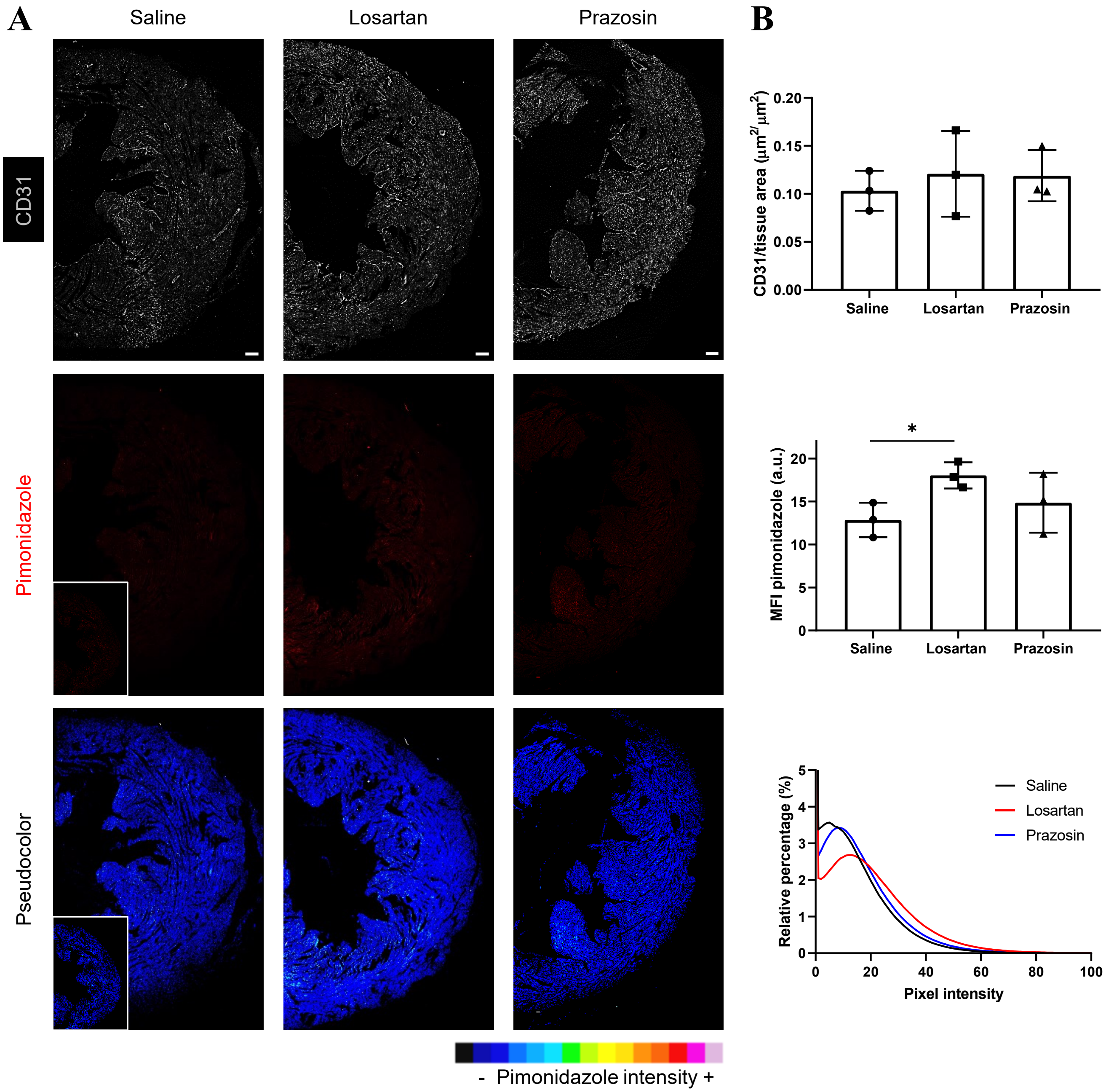

Figure S5 (related to Figure 4). Longer-term effect of the vasodilators losartan and prazosin on oxygenation of neonatal mouse hearts. A, Confocal microscopy MIP of hearts from mice treated with saline, losartan or prazosin from P2 to P6, sacrificed at P14 and stained for CD31 (grey) and pimonodazole (red); pseudocolor image from hypoxiprobe after conversion to 8-bitmap images in ImageJ is also shown. Insets, negative control without primary antibody. Scale bar, 100 µm. **B**, Bar graphs of the quantification of the vascular density (CD31+ area per tissue area) and the mean intensity fluorescence (MFI) of the hypoxyprobe and curves of the relative frequency of hypoxyprobe signal. Bar graphs show individual mouse values and means ± S.D. Data were compared by unpaired two-tailed Student t-test versus saline control, $*p \leq 0.05$.

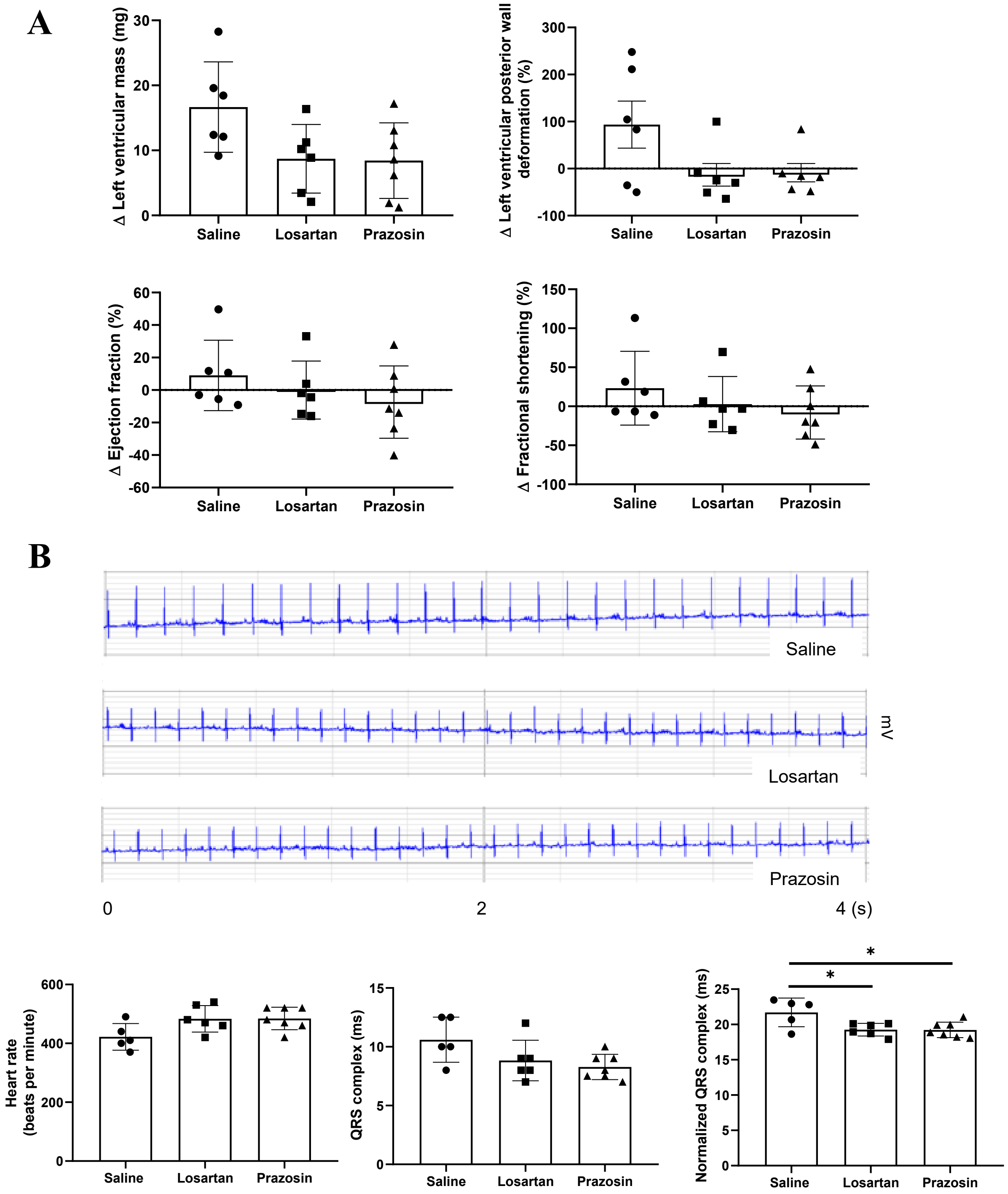

Figure S6 (related to Figure 5). Reduced capillary pruning by vasodilator treatment leads to immature cardiac contractility and conductance. A, Quantification by echocardiogram of left ventricular mass, left ventricular posterior wall deformation, ejection fraction and fractional shortening in P7 mice (treated with saline or low-dose losartan or prazosin from P2 to P6) compared to those parameters in non-treated P1 mice. **B**, Representative electrocardiographic recordings and quantification of heart rate and QRS complex interval (and also QRS normalized for heart rate) in P7 mice treated as in A.

	Saline	Losartan (10 mg/kg)	Losartan (25 mg/kg)	Prazosin (10 mg/kg)	Prazosin (25 mg/kg)
**Fractal-Based Metrics**					
1- Fractal Dimension	1.880 ± 0.032	1.886 ± 0.035	**1.857 ± 0.033*#**	1.894 ± 0.035	1.875 ± 0.028
2- Lacunarity	0.667 ± 0.034	0.671 ± 0.046	**0.698 ± 0.028*#**	**0.642 ± 0.031***	0.665 ± 0.011
3- Succolarity (x10^{-5})	41.89 ± 9.59	44.81 ± 10.02	**35.30 ± 7.28*#**	**47.42 ± 11.27***	**38.84 ± 9.23#**
**Minkowski-Based Metrics**					
4- Vascular Volume Density (%)	16.26 ± 3.18	17.19 ± 2.17	**13.11 ± 1.60*#**	**18.29 ± 2.57***	**13.90 ± 2.78*#**
5- Surface Area Density (μm^2/μm^3)	0.146 ± 0.018	0.149 ± 0.015	**0.129 ± 0.020*#**	**0.165 ± 0.016***	**0.137 ± 0.031#**
6- Breadth Density (x10^{-3}) (μm/μm^3)	4.59 ± 0.97	4.39 ± 0.77	4.44 ± 0.93	4.98 ± 1.23	4.61 ± 1.11
7- Euler-Poincaré Characteristic Density (x10^{-3}) (1/μm^3)	-5.2 ± 1.46	-5.07 ± 1.15	-4.68 ± 2.14	**-6.98 ± 2.14***	**-5.38 ± 2.93#**
**Graph-Based Metrics**					
7- Vascular length density (μm/μm^3) (x10^3)	11.9 ± 2.1	11.93 ± 1.72	**10.58 ± 2.29*#**	**14.08 ± 2.40***	**11.66 ± 3.63#**
8- Vascular surface density (μm^2/μm^3)	0.125 ± 0.021	0.129 ± 0.017	**0.108 ± 0.020*#**	**0.147 ± 0.019***	**0.117 ± 0.033#**
9- Vascular segment diameter (μm)	3.37 ± 0.26	**3.53 ± 0.27***	**3.27 ± 0.17#**	3.39 ± 0.25	**3.23 ± 0.11*#**
10- Vascular volume density (%)	11 ± 2	11.8 ± 1	**9.2 ± 1*#**	**12.9 ± 1***	**9 ± 2#**
11- Vascular segment surface (μm^2)	128 ± 19	136 ± 22	124 ± 21	**115 ± 19***	119 ± 24
12- Vascular segment volume (μm^3)	113 ± 23	125 ± 27	**106 ± 21#**	102 ± 22	100 ± 22
13- Tortuosity (μm/μm)	1.439 ± 0.04	**1.420 ± 0.02***	1.427 ± 0.03	**1.416 ± 0.02***	1.430 ± 0.03
14- Vascular segments / mm^3 of tissue (x10^4)	100.75 ± 27.53	98.30 ± 24.82	92.23 ± 36.24	**134.5 ± 40.41***	**109.33 ± 56.38#**
15- Vascular segments / mm of vascular length	82.79 ± 9.94	81.36 ± 9.61	84.64 ± 13.84	**93.76 ± 12.12***	88.50 ± 18.02
16- Vascular segment length (μm)	12.25 ± 1.49	12.46 ± 1.49	12.09 ± 1.78	**10.84 ± 1.78***	11.73 ± 2.24
17- Vessels of diameter <5 μm (%)	95.9 ± 3.6	94.5 ± 4.4	**98.3 ± 1.5*#**	96.15 ± 3.4	**98.8 ± 1.00*#**
18- Vessels of diameter between 5 and 8 μm (%)	3.96 ± 3.59	5.35 ± 4.35	**1.68 ± 1.52*#**	3.8 ± 3.4	**1.19 ± 0.98*#**
19- Vessels of diameter >8 μm (%)	0.06 ± 0.19	0.06 ± 0.17	0.004 ± 0.01	0.05 ± 0.11	0.005 ± 0.02
20- Branching nodes / mm^3 of tissue (x10^4)	59.77 ± 16.42	58.53 ± 14.61	54.45 ± 21.67	**79.57 ±23.56***	**64.19 ± 33.08#**
21- Branching nodes / mm vascular length	49.32 ± 6.14	48.68 ± 5.78	50.24 ± 8.46	**55.84 ± 7.18***	52.32 ± 10.88
22- Node complexity	2.77 ± 0.10	2.79 ± 0.09	2.73 ± 0.07	**2.85 ± 0.06***	**2.77 ± 0.13#**
23- Number of bifurcations	1198 ± 218	1193 ± 316	1091 ± 234	**1485 ± 256***	1332 ± 476
24- Number of trifurcations	197 ± 56	195 ± 77	181 ± 56	**272 ± 67***	242 ± 119
25- Blind-ends / mm^3 of tissue (x10^3)	19.78 ± 7.33	18.57 ± 7.91	20.49 ± 6.92	21.34 ± 7.26	**25.25 ± 6.81***
26- Blind-ends / mm of vascular length	1.66 ± 0.71	1.49 ± 0.61	**2.035 ± 0.86#**	1.45 ± 0.54	**2.33 ± 1.072*#**
**Efficiency in oxygen diffusion**					
27- Diffusion distance (μm)	5.21 ± 0.51	5.20 ± 0.37	**5.57 ± 0.55*#**	**4.80 ± 0.38***	**5.40 ± 0.79#**
28- Median extravascular distance (μm) (Frequency distribution)	8.55 ± 1.54	8.08 ± 1.24	8.48 ± 1.17	**7.63 ± 1.34***	**9.02 ± 1.67#**
29- Maximal extravascular distance (μm) (Frequency distribution)	15.66 ± 2.9	14.77 ± 2.3	15.54 ± 2.2	**13.91 ± 2.5***	**16.55 ± 3.1#**
30- Median extravascular distance (μm) (All vessels)	2.91 ± 0.3	2.86 ± 0.2	**3.28 ± 0.3*#**	**2.62 ± 0.2***	**3.15 ± 0.5*#**
31- Maximal extravascular distance (μm) (All vessels)	7.01 ± 0.7	6.90 ± 0.6	**7.64 ± 0.7*#**	**6.40 ± 0.4***	**7.48 ± 1.05*#**
32- Intercapillary distance (μm)	10.84 ± 1.5	11.76 ± 2.3	11.29 ± 1.2	10.84 ± 1.5	10.65 ± 1.3
**Endothelial cell density-related metrics**					
33- Endothelial cells / mm^3 of tissue (x10^5)	4.12 ± 0.8	3.95 ± 1.2	3.88 ± 0.7	4.58 ± 0.8	3.99 ± 1.0
34- Endothelial cells / mm^3 vascular volume (x10^6)	2.76 ± 0.7	2.65 ± 0.6	2.97 ± 0.4	2.55 ± 0.6	2.86 ± 0.4
35- Endothelial cells / mm vascular length	35.33 ± 4.41	34.72 ± 4.78	37.12 ± 3.77	**30.84 ± 2.65***	**34.86 ± 4.42#**

Table S1. 3D angio-architecture of the postnatal coronary microvasculature. Parameters extracted with the segmentation and skeletonization algorithm described in Gkontra et al., 2018. Data represent means ± S.D. n= 8, 9, 5, 8, 5 mice treated with saline, losartan (10 and 25 mg/kg/day) and prazosin (10 and 25 mg/kg/day), respectively, analyzed in 8 independent experiments. Values were analyzed by one-way ANOVA with Tukey's post-hoc corrections for multiple testing of the two drug doses versus saline. Parameters that are significantly increased (**red**) or decreased (**blue**) compared to control are indicated by * and compared to the corresponding low dose of drug in bold and by #.

	P1			P7		
**Parameters**	**Saline**	**Losartan**	**Prazosin**	**Saline**	**Losartan**	**Prazosin**
**Ecography**						
1- Anterior wall thickness (mm) (Systole)	0.49 ± 0.10	0.58 ± 0.12	0.56 ± 0.11	0.83 ± 0.14	0.74 ± 0.08	0.68 ± 0.13
2- Anterior wall thickness (mm) (Diastole)	0.34 ± 0.05	0.30 ± 0.07	0.34 ± 0.09	0.50 ± 0.13	0.46 ± 0.09	0.43 ± 0.11
3- Left ventricular posterior wall thickness (mm) (Systole)	0.51 ± 0.04	0.57 ± 0.12	0.54 ± 0.09	0.83 ± 0.17	0.72 ± 0.06	0.69 ± 0.13
4- Left ventricular posterior wall thickness (mm) (Diastole)	0.31 ± 0.06	0.27 ± 0.05	0.33 ± 0.06	0.42 ± 0.12	0.40 ± 0.03	0.43 ± 0.06
5- Left ventricular internal diameter (mm) (Systole)	0.75 ± 0.18	0.61 ± 0.17	0.71 ± 0.20	0.97 ± 0.34	0.87 ± 0.30	1.10 ± 0.42
6- Left ventricular internal diameter (mm) (Diastole)	1.27 ± 0.12	1.26 ± 0.24	1.36 ± 0.29	1.86 ± 0.22	1.72 ± 0.44	1.86 ± 0.33
7- Anterior wall deformation (%)	44.3 ± 26	105.5 ± 79	67.0 ± 31	75.2 ± 37	66.6 ± 38	64.8 ± 36
8- Left ventricular posterior wall deformation (%)	69.5 ± 35	113.2 ± 50	66.1 ± 43	99.4 ± 39	79.1 ± 18	61.5 ± 24
9- Left ventricular mass (mg)	5.50 ± 1.8	4.48 ± 1.2	6.46 ± 2.0	14.33 ± 4.4	13.20 ± 4.6	14.89 ± 5.1
10- Left ventricular volume (mm^3) (Systole)	1.06 ± 0.55	0.63 ± 0.40	0.96 ± 0.67	2.38 ± 1.83	1.74 ± 1.4	3.4 ± 3.48
12- Left ventricular volume (mm^3) (Diastole)	3.99 ± 1.00	4.15 ± 2.0	5.07 ± 2.51	10.92 ± 3.1	9.61 ± 5.6	11.13 ± 5.1
13- Ejection fraction (%)	74.1 ± 12.3	83.7 ± 10.0	81.5 ± 9.7	79.7 ± 14.0	82.6 ± 8.9	74.4 ± 13.9
14- Fractional shortening (%)	40.9 ± 11.9	50.7 ± 12.3	47.7 ± 9.3	48.4 ± 15.1	49.6 ± 10.3	42.3 ± 12.6
13- Coronary Flow rate (cm/s) (Systole)	57.3 ± 34.6	35.8 ± 15.4	38.3 ± 10.4	40.6 ± 10.0	53.7 ± 16.7	57.5 ± 20.5
15- Coronary Flow rate (cm/s) (Diastole)	188.3 ± 102	132.7 ± 44	144.7 ± 48	208 ± 54	188 ± 48	219 ± 49
16- Coronary Flow Ratio (Systole/Diastole)	0.30 ± 0.09	0.27 ± 0.10	0.27 ± 0.04	0.20 ± 0.05	0.29 ± 0.09	0.25 ± 0.06
17- Coronary Diameter (mm)	0.093 ±0.04	0.107 ± 0.02	0.132 ± 0.02	0.134 ± 0.02	0.134 ± 0.02	0.149 ± 0.04
**Electrocardiogram**						
18- P wave (ms)				10.70 ± 1.44	11.08 ± 1.68	9.78 ± 1.35
19- PR interval (ms)				38.40 ± 3.41	37.17 ± 3.65	37.71 ± 3.53
20- QRS complex (ms)				10.60 ± 1.91	8.83 ± 1.72	8.28 ± 1.075
21- QT interval (ms)				54.70 ± 6.55	51.75 ± 7.1	47.86 ± 12.77
22- Normalized QRS complex (ms)				21.71 ± 2.02	**19.27 ± 0.89***	**19.23 ± 1.1***
23- Normalized QT interval (ms)				54.83 ± 6.55	51.88 ± 7.10	51.63 ± 5.78

Table S3. Echocardiogram and electrocardiogram analyses in vasodilator-treated mouse neonates. Parameters obtained by ultrasound and electrocardiogram of P1 (non-treated) and P7 (treated with saline or low dose losartan or prazosin from P2 to P6) mice. Data represent the means ± S.D. from n= 6, 6, 7 mice treated with saline, losartan (10 mg/Kg/day) and prazosin (10 mg/Kg/day), respectively, analyzed in 3 independent experiments. Values were analyzed by one-way ANOVA with Tukey's post-hoc corrections for multiple testing and those that are significatively decreased compared to control are indicated in **blue** and by *.
